# Disconnect To Reconnect: How Variations between Types of Smartphone Bans Influence Students’ Well-being and Social Connectedness in Dutch Secondary Education

**DOI:** 10.1007/s10964-025-02313-6

**Published:** 2026-01-07

**Authors:** Elien Vanluydt, Regina van den Eijnden, Lisanne Vonk, Polina Putrik, Thérèse van Amelsvoort, Philippe Delespaul, Mark Levels, Tim Huijts

**Affiliations:** 1 Research Centre for Education and the Labour Market (ROA), School of Business and Economics, Maastricht University, P.O. Box 616, Maastricht, 6200 MD the Netherlands; 2 Interdisciplinary Social Science, University Utrecht, Heidelberglaan 8, 3584 CS Utrecht, The Netherlands; 3 Expertise group Child Health, Netherlands Organisation for Applied Scientific Research (TNO), Sylviusweg 71, 2333 BE Leiden, the Netherlands; 4 Department of Social Medicine, Care and Public Health Research Institute (CAPHRI), Faculty of Health Medicine and Life Sciences (FHML), Maastricht University, P.O. Box 616, 6200 MD Maastricht, the Netherlands; 5 Department of Knowledge & Innovation, Living Lab Public Health Mosa, Public Health Service South Limburg (GGD Zuid Limburg), P.O. Box 33, 6400 AA Heerlen, the Netherlands; 6 Department of Psychiatry & Neuropsychology, Mental Health and Neuroscience Institute, Maastricht University, P.O. Box 616, 6200 MD Maastricht, the Netherlands; 7 Mondriaan Mental Health Centre, P.O. Box 4436, 6401 CX Heerlen, the Netherlands

**Keywords:** Smartphone ban policies, Problematic smartphone use, Well-being, Social connectedness, Bullying

## Abstract

Smartphone bans are gaining popularity in education, with approximately 40% of countries currently implementing such policies. Some schools apply smartphone restrictions to the classroom only (partial bans), while others extend the restrictions to the whole school grounds (full bans), hoping to foster student well-being and strengthen social connectedness at school. However, there is currently no empirical evidence that stricter policies are more effective in achieving these intended benefits. The current study examined how variations in type of ban affect adolescents’ screentime, problematic social media use, well-being, social connectedness at school, and bullying. The sample consisted of Dutch adolescents from 24 schools (9 partial-ban schools and 15 full-ban schools) who participated in the 2024–2025 EPoSS Study (*N* = 1398; *M*_age_ = 16.2; *SD* = 1.2; 51.5% were female; 38.3% were in partial-ban schools and 61.7% in full-ban schools). No significant differences were found for any of the well-being or bullying outcomes. However, full bans were associated with lower student-teacher connectedness and, for girls, reduced school belonging. These findings indicate that stricter bans do not yield the intended benefits for students’ well-being or bullying and may even undermine students’ social connectedness at school.

**Supplementary Information:**

The online version contains supplementary material available at 10.1007/s10964-025-02313-6.

## Introduction

Concerns are growing about the impact of increased smartphone use on adolescents’ cognitive (Paterna et al., 2024) and socioemotional development (Sohn et al., 2019). In response to these concerns, smartphone bans are being implemented worldwide in schools (UNESCO, 2023). For example, The Netherlands introduced a smartphone ban in secondary schools in January 2024. Despite growing international interest in smartphone bans at schools, there is limited robust evidence supporting their effectiveness (Campbell et al., 2024). This partly stems from the variations in implementation, ranging from informal classroom rules to strict school ground policies. This variation complicates comparisons between schools and highlights the relevance of examining the scope and degree of restrictions. Moreover, while most studies focus on academic achievement when considering smartphone bans, this narrow view overlooks other key aspects of student development like well-being (Bas, 2021) and social connectedness at school (Delgado et al., 2015). This study will not merely focus on the effectiveness of smartphone bans in general, but rather on how variations between types of smartphone bans (i.e., classroom only or whole school grounds) influence secondary school students’ well-being and social connectedness at school.

### Negative Associations between Problematic Smartphone Use and Outcomes

Problematic smartphone use refers to addiction-like symptoms, such as restlessness or stress when deprived of access, or neglect of responsibilities (Pivetta et al., 2019). While this can involve various activities (e.g., social media, gaming, video watching), problematic social media use specifically concerns addiction-like symptoms related to social media. Both differ from general smartphone use, which is typically defined by usage intensity alone (Shannon et al., 2022).

Extensive evidence links problematic smartphone use to several distinct negative outcomes. Problematic smartphone use shows a small negative association with students’ academic achievement, and is therefore considered a potential determinant of decreased academic achievement (Paterna et al., 2024). This negative link might be explained by the time trade-off between smartphone use and studying (Baert et al., 2020), as well as the cognitive overload and reduced concentration that can result from frequent switching between academic and social activities on devices (Aru & Rozgonjuk, 2022). Beyond academic outcomes, problematic social media use is associated with higher odds of depression, anxiety, stress, and reduced sleep quality, supporting a negative association between problematic social media use and well-being (Sohn et al., 2019). Similarly, several studies report negative associations between problematic smartphone use and aspects of social connectedness at school, such as student-teacher connectedness (Shi et al., 2022) and classmate connectedness (Wang et al., 2017). One potential mechanism underlying these associations is that supportive school relationships foster higher self-esteem, which in turn reduces the likelihood of problematic smartphone use (Wang et al., 2017). Finally, there is a positive association between problematic smartphone use and bullying victimization at school (Saied et al., 2022). In contemporary educational settings, attention must be given to both in-person bullying interactions that occur at school and to bullying that occurs online, which is referred to as cyberbullying. Evidence on the link between problematic smartphone use and cyberbullying, however, is mixed. Some studies find direct associations between problematic smartphone use and cyberbullying victimization (Craig et al., 2020) and perpetration (Kırcaburun et al., 2019), whereas others find no association with either (Blinka et al., 2022). Taken together, these findings support concerns raised by educational stakeholders about the potential harms of smartphones at school.

### Smartphone Bans in Schools

In light of increasing concerns about the detrimental effects of problematic smartphone use on students, more and more policymakers and schools are introducing smartphone bans in the classroom or even the entire school grounds. Around 40% of countries in the world (e.g., the Netherlands, Belgium, France, Israel, Turkey, Bangladesh, the United States, and certain areas in Canada and Australia) have enacted laws restricting smartphone use during school hours (UNESCO, 2023). Despite this trend, evidence on the effectiveness of smartphone bans remains limited and mixed (for a review of the literature, see Campbell et al., 2024).

The majority of studies evaluating smartphone bans focused on academic achievement. While there are some studies that report an increase in academic performance after implementation of smartphone bans (e.g., Abrahamsson, 2024), there are almost an equal number of studies that report no differences in academic performance irrespective of bans (e.g., Kessel et al., 2020). It could be argued that there seems to be a negative although small impact of smartphone bans on academic outcomes, but only in certain circumstances for certain students (Campbell et al., 2024). The only study to date to investigate smartphone bans in schools in relation to students’ screentime and problematic social media use, indicates that students’ screentime after school increases to compensate for restrictions during school hours, and found no differences for problematic usage (Goodyear et al., 2025). Empirical findings for well-being outcomes are also scarce and mixed: Two studies found no significant effects of smartphone bans at school on students’ well-being (Guldvik & Kvinnsland, 2018; Goodyear et al., 2025), while another reported reduced mental health care needs for girls only (Abrahamsson, 2024). No studies have investigated whether and how smartphone bans influence adolescents’ social connectedness at school. Finally, while there is emerging evidence that bullying at school seems to decrease after implementation of smartphone restrictions, two older studies show that cyberbullying was more frequent in schools with restrictions in place (Davis & Koepke, 2016; Walker, 2013).

The mixed evidence may partly stem from the varied ways smartphone bans are implemented in practice. The term “ban” can refer to anything from informal classroom rules to strict school-wide policies, blurring the line between schools with and without bans. This makes direct comparisons difficult and highlights the need to examine different types of bans. Currently, there is only one study comparing the effectiveness of these various types of smartphone bans in the UK (Goodyear et al., 2025), finding no differences between different types of bans for several different outcomes of mental health, physical activity, disruptive classroom behaviour and problematic social media use. More studies that focus on the degree and scope of restrictions, rather than their mere presence, are needed and may offer more nuanced insights into their effects on students and help explain mixed findings in previous research.

Moreover, the majority of studies focused on the impact of smartphone bans on academic performance, often seen as education’s primary goal. However, this narrow focus overlooks other key aspects of student development, such as well-being (Bas, 2021) and social connectedness at school (Delgado et al., 2015). As such, smartphone ban policies should be evaluated on more than the potential impact on academic performance, but also with consideration for their broader effects on students’ well-being and social connectedness at school.

Finally, inconsistent findings on smartphone bans may partly reflect sex-specific responses. For instance, smartphone restrictions reduced the need for mental health care, but only for girls (Abrahamsson, 2024). This suggests a differential impact, possibly rooted in the distinct mental health challenges typically experienced by boys and girls. Girls are more prone to internalizing symptoms like anxiety and depression, while boys more often exhibit externalizing behaviours (Buil et al., 2017). Another explanation may be the higher rates of problematic social media use among girls (Boer et al., 2022). These differences may shape students’ responses to smartphone restrictions and explain mixed findings in prior research. Further investigation into these gendered effects is warranted.

## Current Study

Often smartphone ban policies lack clarity on how to implement and execute the smartphone ban. In the Netherlands, for example, school boards were given autonomy to establish their own agreements, which has led to variations among schools: some schools have restricted the ban to the classroom only (i.e., a partial smartphone ban), while others extended the prohibition to the entire school grounds (i.e., a full smartphone ban). Importantly, the current literature offers only limited evidence to guide decisions on which type of ban to implement. As a result, school boards are adopting policies without clear direction, highlighting the urgent need for empirical studies, like the current study, that directly compare the outcomes of partial and full bans to support evidence-based policymaking. More specifically, the current study aims to address the gap in the literature regarding the effectiveness of various *types* of smartphone bans in secondary school, focusing on well-being and social connectedness at school as outcomes. In addition to extending previous research by examining different types of bans, this study also considers outcomes that have been largely overlooked in prior evaluations of smartphone ban policies, specifically those related to social connectedness at school. Furthermore, it examines potential gender differences, thereby providing a more nuanced understanding of how such policies may differentially affect students. Two research questions were formulated: To what extent does the type of smartphone ban (i.e., partial versus full) in secondary schools influence problematic social media use and screentime, well-being, social connectedness at school, bullying at school, and cyberbullying; and are these associations moderated by sex? It is hypothesized that overall outcomes will be more favorable in schools with a full smartphone ban compared to those with a partial ban. Moreover, differential associations with well-being outcomes are expected for boys and girls.

## Methods

### Participants and Procedure

The current study used data from the EPoSS project (Early Predictors of School Success) that investigates the relationships between physical, mental, and social health, and academic achievement in a large sample of primary and secondary school students in the Netherlands. Data collection occurred between September 2024 and February 2025 in 27 secondary schools in six out of twelve different geographical regions of the Netherlands. Within the participating schools, all students were invited to participate if one parent/caregiver gave informed consent or students were above 16 years. All students provided active informed consent. Students in grade 9, 10, 11, and 12 completed a digital survey during school hours, which took approximately 30 minutes. Additionally, another survey regarding several school policies was filled out by one school employee (i.e., rector, vice-rector, teacher, school psychologist, etc.). An English translation of the relevant survey questions is included in Appendix A. In total, 1950 student-surveys were collected, of which 509 were excluded due to lack of consent, incorrect grade, empty surveys, or duplicates. One school with only a single participating student was excluded. Additionally, two schools that had not yet implemented a smartphone ban were excluded from the study (43 student-surveys). The sample size in this condition was significantly smaller and less diverse compared to the other groups, which would have introduced bias in the comparisons. Moreover, the responses from these schools might not be reliable, as all schools were obligated to have a smartphone ban in place at the time of assessment. The final sample consisted of 1398 participants from 24 schools (678 boys, 687 girls, and 33 students who preferred not to disclose their sex at birth) with an average age of 16.2 years (*SD* = 1.2 years). Statistics Netherlands (CBS) securely stores the encrypted data and facilitates individual linkage with the Netherlands Cohort Study on Education (NCO; Haelermans et al., 2020). Information on parental educational attainment, migration background, school size and school socio-economic composition was used from the latest NCO dataset (2022–2023).

### Measures

#### Type of Smartphone Ban

In the school survey filled out by one school employee (i.e., rector, vice-rector, teacher, school psychologist, etc.), a question about smartphone ban policies was included: “At your school, is there a smartphone ban in the classroom only or across the entire school?” Answer options were: “Yes, in the classroom only”, “Yes, in the classroom and the entire school”, “Yes, but different … [open text box to fill in]” and “No, we do not (yet) have a smartphone ban in our school.” Schools that did not yet have a smartphone ban (*N* = 2) were excluded from the study. All schools that explained their type of ban in the open text box (*N* = 4) could be reclassified to either a classroom only ban or an entire school ban. Hence, a categorical variable with two categories remains: Partial ban (i.e., classroom only) and Full ban (i.e., entire school ban).

#### Problematic Social Media Use 

The Problematic Social Media Use Scale (see Vonk et al., 2025 for a more detailed description) includes eight items about difficulties in reducing social media usage, external suggestions to limit social media time, preferring social media over face-to-face interactions, feeling restless, stressed, or irritated when (not) using social media or their phone, neglecting other tasks like homework in favour of social media, and using social media when feeling bad. Responses were rated on a five-point scale from never (0) to very often (4). The average score across all items was calculated, with scores ranging from 0 to 4. Higher scores indicate greater problematic social media use. Cronbach’s alpha was 0.82, indicating good internal consistency.

#### Screentime

Screentime was measured using a single item: “How many hours per day do you spend behind the computer, tablet, smartphone or television in your free time (so not for school)?” Responses were rated on a five-point Likert scale: “(almost) never”, “one hour per day”, “two hours per day”, “three hours per day”, and “four hours or more per day”. Responses were dichotomized to distinguish between below-average and above-average screentime (Qi et al., 2023): three or more hours per day was coded as 1, less than three hours was coded as 0.

#### Well-Being

Well-being was measured as a broad construct and included a general life satisfaction measure, a loneliness measure, and questions about 12 different psychosomatic complaints. General life satisfaction was measured by asking students to provide a rating of their current life on a scale ranging from 0 (“worst possible life”) to 10 (“best possible life”). This measure captures a wide range of well-being, from very negative to very positive. The construct validity of this continuous outcome for primary school children is well-supported (Huebner, 2004). Loneliness was measured by one question: “How often in the last twelve months did you feel lonely?” Students answered on a five-point Likert scale with response options: “never”, “almost never”, “sometimes”, “often” and “always.” Psychosomatic complaints were measured by the frequency of 12 symptoms (i.e., headache, stomachache, backache, feeling unhappy, irritated, nervous, dizzy, nauseous, difficulty concentrating, difficulty falling asleep, poor sleep quality, and daytime fatigue). Students reported how often they experienced each complaint over the past six months using a five-point Likert scale: “almost never or never,” “almost every month,” “almost weekly,” “more than weekly,” and “almost every day.” Each complaint was then recoded into a binary variable, where a value of 1 indicates experiencing the complaint more than weekly. This binary classification is commonly used in psychosomatic research, as experiencing such complaints on a near-daily basis reflects a substantially different level of severity and clinical relevance compared to occasional or monthly occurrences (Haugland & Wold, 2001). These questions and the binary classification are also used in the Health Behaviour in School-aged Children (HBSC) study (Boer et al., 2022). Confirmatory Factor Analysis showed good fit for a three-factor model comprising Physical Complaints, Emotional Complaints and Fatigue as latent variables (CFI = 0.95, TLI = 0.94, RMSEA = 0.07, SRMR = 0.08). The three latent constructs showed good composite reliability: Physical Complaints (CR = 0.88), Emotional Complaints (CR = 0.84), and Fatigue (CR = 0.90). See appendix B for full model information. The latent constructs served as the primary outcomes in subsequent analyses.

#### Social Connectedness at School

Three aspects of social connectedness at school were assessed: school belonging, student-teacher connectedness, and classmate connectedness. School belonging describes “the extent to which students feel personally accepted, respected, included, and supported by others in the school social environment”(Goodenow, 1993, pp. 60–61). This concept was assessed using eight statements (e.g., “I feel safe at school,” “I feel lonely at school”), each rated on a five-point Likert scale from 1 (“I completely agree”) to 5 (“I completely disagree”). Positively phrased items were reverse coded, and an average school belonging score was calculated for each student, ranging from 1 to 5, with 1 indicating the lowest sense of belonging and 5 indicating the highest. The school belonging scale demonstrated good reliability, with a Cronbach’s alpha of 0.99. Student-teacher connectedness refers to how students perceive their teachers as caring, respectful, and attentive (García-Moya et al., 2018). The survey included three positively phrased statements (e.g., “My teacher accepts me the way I am,” “I have a lot of trust in my teachers”), each rated on a five-point Likert scale from 1 (“completely agree”) to 5 (“completely disagree”). The items were reverse-coded and an average score was calculated, ranging from 1 to 5, with 1 indicating the lowest level of student-teacher connectedness and 5 indicating the highest. The student-teacher connectedness scale demonstrated good reliability, with a Cronbach’s alpha of 0.97. Classmate connectedness refers to how students perceive their classmates as caring, respectful, and willing to listen to one another. The survey included three positively phrased statements (e.g., “My classmates are friendly and helpful,” “My classmates accept me as I am”), each rated on a five-point Likert scale from 1 (“completely agree”) to 5 (“completely disagree”). All items were reverse-scored, and an average score was calculated, ranging from 1 to 5, with 1 indicating the lowest level of classmate connectedness and 5 indicating the highest. The classmate connectedness scale demonstrated good reliability, with a Cronbach’s alpha of 0.96.

#### Bullying at School and Cyberbullying

Four questions about bullying were included in the survey. These questions are also used in the HBSC study (Boer et al., 2022). Two questions assessed bullying at school (victimization: “How often were you bullied at school in the last few months?” and perpetration: “How often did you participate in bullying someone else at school in the last few months?”) and another two questions assessed cyberbullying (victimization: “How often were you bullied online (cyberbullying) in the last few months?” and perpetration: “How often did you participate in bullying someone else online (cyberbullying) in the last few months?”). Each question was rated on a five-point Likert scale with response options: “This didn’t happen in the last few months”, “It happened once or twice”, “Two or three times a month”, “Approximately once a week”, or “A few times a week.” All four variables are included as separate outcomes in the analyses (i.e., bullying at school victimization, bullying at school perpetration, cyberbullying victimization, and cyberbullying perpetration).

#### Covariates

Five student-level covariates were included: sex at birth (male/female; prefer not to say was recoded to missing), age (in years), parents’ highest educational attainment (low/medium/high; following Standard Education Classification guidelines from Statistics Netherlands; see CBS, 2021), migration background (i.e., being a first or second-generation migrant; yes or no), and educational track (vmbo, havo or vwo). The Dutch secondary education system is divided into three main educational tracks (for a more detailed description see Jacobs et al., 2023). The first track includes lower vocational, higher vocational, and lower academic education (vmbo in Dutch), which takes four years to complete and leads to upper secondary vocational education and training. The second track, medium academic education (havo in Dutch), lasts five years and provides access to universities of applied sciences. The third and highest track, high academic education (vwo in Dutch), spans six years and prepares students for research universities.

Grade (9, 10, 11, 12) was excluded as a student-level covariate due to high multicollinearity with age and track, as indicated by a variance inflation factor (VIF) greater than 10. This is partly due to the way data collection was structured. Data was collected in Grade 9 and the exam classes (Grade 10, 11, and 12), which are different years for each educational track (i.e., Grade 10 for vmbo, Grade 11 for havo, and Grade 12 for vwo).

The first covariate at the school-level is the school’s socio-economic composition. To quantify this, a disadvantage score was computed for each student using a weighted algorithm developed by the Dutch Ministry of Education, incorporating parental education level, migration background, duration of residence in the Netherlands, and neighbourhood socio-economic indicators. The school-level score was calculated as the average of all individual scores, serving as a proxy for the overall socio-demographic profile of the school. A key strength of this measure is its ability to capture multiple correlated dimensions of disadvantage within a single composite score (also see Bluemink et al., 2024). The second school-level covariate was school size, operationalized as a binary variable indicating whether a school enrolled more than 750 students (classified as large) or not. Urbanisation and denomination were considered as covariates. However, urbanicity was not included due to high multicollinearity with school socio-economic composition and school size, as indicated by a variance inflation factor (VIF) greater than 10. Denomination was not included because several categories lacked sufficient representation for reliable estimation.

### Statistical Analysis

All analyses were conducted using R (Version 3.6.0 or later; Posit Team, 2024). Missing data (see Appendix C) were handled using multiple imputation with five datasets and 30 iterations via the *mice* package (van Buuren & Groothuis-Oudshoorn, 2011). Statistical significance was set at α = 0.05.

To examine the relationship between the independent variable (i.e., type of smartphone ban) and outcomes (i.e., problematic smartphone use, screentime, well-being, social connectedness at school and bullying), multilevel regression analyses were performed. Given the nested structure of the data (students within schools), models included two levels: students (Level 1) and schools (Level 2). Fixed effects included the primary predictor (i.e., type of smartphone ban) and covariates (see Sect. 2.1.7), with random intercepts and slopes at the school level.

Each outcome was analysed using three nested models:

- Model 1 (baseline model) included only the primary predictor (type of smartphone ban).
- Model 2 extended model 1 by adding student-level covariates.
- Model 3 extended model 2 by adding school-level covariates.

Linear models were used for continuous outcomes; logistic models with odds ratios (OR) for binary outcomes. All models were estimated using maximum likelihood estimation. Model performance was assessed using marginal and conditional R², and intraclass correlation coefficients (ICCs) were reported. In the results, only the final models (Model 3) are reported, as adding covariates changed the results for only one outcome (student-teacher connectedness) from significant to marginal. Full model results (Model 1–3) are available in Appendix D.

To examine whether the relationships between type of smartphone ban and the outcomes differed by sex, the models (i.e., Model 3) were stratified by sex, estimating separate models for male and female students. This approach allowed us to assess moderation by sex without overcomplicating the models with interaction terms.

## Results

Table 1 presents descriptive statistics for student- and school-level covariates, both for the total sample and separately by smartphone ban type (partial vs. full). Table 2 presents descriptive results for all outcomes, again both for the total sample and separately by smartphone ban type.

**Table 1:** Student-level covariates (*n* = 1398) and school-level covariates (*n* = 24)

Student-level covariates	Categories	Total sample % (*n* = 1398)	Partial SPB^a^ % (*n* = 535)	Full SPB^a^ % (*n* = 863)
Sex	male	49.9	54.4	47.2
	female	50.1	45.6	52.8
Grade	grade 9	35.8	25.4	42.3
	grade 10	16.7	22.2	13.3
	grade 11	18.2	23.9	14.6
	grade 12	29.3	28.4	29.8
Educational track	vmbo	25.8	34.0	20.6
	havo	28.5	34.8	24.6
	vwo	45.8	31.2	54.8
Highest parental educational attainment	low	8.8	10.8	7.5
	mid	21.0	23.6	19.4
	high	70.2	65.6	73.1
Migration background	yes	29.6	26.9	31.3
	no	70.2	73.1	68.7
		Mean (*SD*)	Mean (*SD*)	Mean (*SD*)
Age (years)		16.2 (1.4)	16.4 (1.3)	16.1 (1.5)

School-level covariates	Categories	Total sample % (*n* = 24 )	Partial SPB^a^% (*n* = 9)	Full SPB^a^ % (*n* = 15)
School socio-economic composition	[2,25] percentile	29.2	33.3	26.7
	(25,50] percentile	12.5	0.0	20.0
	(50,75] percentile	29.2	22.2	33.3
	(75,100] percentile	29.2	44.4	20.0
School size	small – mid	54.2	66.7	46.7
	large	45.8	33.3	53.3
Urbanization	(Very) small-average	29.2	44.4	20.0
	Strong-very strong	70.8	55.6	80.0
Denomination	protestant-catholic	12.5	22.2	6.7
	roman-catholic	16.7	44.4	0.0
	independent	29.2	11.1	40.0
	other	20.8	11.1	26.7
	public	20.8	11.1	26.7

^**a**^ SPB = smartphone ban

**Table 2:** Mean, standard deviation (SD) and range for all outcomes

	Total sample (*n* = 1398)			Partial SPB^a^ (*n* = 535)			Full SPB^a^ (*n* = 863)		
Variable	Mean	SD	Range	Mean	SD	Range	Mean	SD	Range
Life satisfaction	7.30	1.47	1–10	7.28	1.44	0–10	7.31	1.50	1–10
Loneliness	2.29	0.97	1–5	2.24	0.99	1–5	2.33	0.96	1–5
Physical complaints^b^	0.08	0.60	-0.60–1.77	0.11	0.60	-0.60–1.77	0.07	0.59	-0.60–1.77
Emotional complaints^b^	0.06	0.62	-0.72–1.62	0.06	0.61	-0.72–1.62	0.06	0.62	-0.72–1.62
Fatigue^b^	0.05	0.61	-0.72–1.44	0.06	0.61	-0.72–1.44	0.05	0.61	-0.72–1.44
Problematic social media use	1.53	0.76	0–4	1.46	0.79	0–4	1.57	0.74	0–4
Screentime	0.45	0.50	0–1	0.50	0.50	0–1	0.42	0.49	0–1
School belonging	3.77	0.66	1–5	3.80	0.66	1–5	3.75	0.66	1–5
Student-teacher connectedness	3.68	0.82	1–5	3.80	0.78	1–5	3.61	0.83	1–5
Classmate connectedness	3.86	0.73	1–5	3.92	0.73	1–5	3.82	0.73	1–5
Bullying at school – victim	1.39	0.93	1–5	1.41	0.96	1–5	1.38	0.91	1–5
Bullying at school – perpetrator	1.48	1.18	1–5	1.50	1.22	1–5	1.46	1.15	1–5
Cyberbullying – victim	1.23	0.78	1–5	1.17	0.67	1–5	1.26	0.84	1–5
Cyberbullying - perpetrator	1.21	0.78	1–5	1.20	0.77	1–5	1.22	0.79	1–5

^**a**^ SPB = smartphone ban; ^b^ latent constructs, for CFA results see appendix B

### Problematic Social Media Use and Screentime

Type of smartphone ban was not significantly associated with problematic social media use: students attending schools with a full smartphone ban experienced equal levels of problematic social media use compared to those in schools with a partial ban. For screentime, we observed a significant association between the type of smartphone ban and the likelihood of reporting high screentime (≥ 3 h/day) in the full sample: students in full-ban schools reported lower odds of high screentime in their free time than students partial-ban schools. However, when stratified by sex, this association did not remain significant. Table 3 presents the results for problematic social media use and screentime in more detail for the full sample and stratified by sex.

### Well-Being

Across all five well-being outcomes (i.e., life satisfaction, loneliness, physical complaints, emotional complaints, and fatigue) there was no significant association with the type of smartphone ban, neither in the full sample nor in the sex-stratified models. Table 4 presents the results for all well-being outcomes for the full sample and stratified by sex.

### Social Connectedness at School

For school belonging, a significant association with the type of smartphone ban was found only among girls: girls attending schools with a full smartphone ban reported lower levels of school belonging compared to their peers attending schools with a partial smartphone ban. No such association was observed in the full sample or among boys. For student-teacher connectedness, there was a marginally significant association with the type of smartphone ban in the full sample. In the sex-stratified models, this association reached conventional standards for statistical significance for both boys and girls: boys and girls attending schools with a full smartphone ban reported lower levels of student-teacher connectedness compared to their peers attending schools with a partial smartphone ban. No significant associations were found between the type of smartphone ban and classmate connectedness in any of the models. Table 5 presents the results of Model 3 for all school connectedness outcomes for the full sample and stratified by sex.

### Bullying at School and Cyberbullying

For all bullying outcomes, there were no significant associations with the type of smartphone ban in either the full sample or the sex-stratified models. Table 6 presents the results of Model 3 for all bullying outcomes for the full sample and stratified by sex.

### The Role of Student- and School-Level Covariates

All student-level covariates were significantly associated with at least one outcome measure. Female students reported higher levels of problematic social media use, loneliness, physical and emotional complaints, and fatigue, and lower levels of life satisfaction, student-teacher connectedness, bullying at school, and cyberbullying. Older students showed lower problematic social media use but were more likely to exceed three hours of daily screentime in their free time. They also reported more emotional complaints and fatigue, alongside stronger school belonging and classmate connectedness. Students in higher educational tracks experienced greater classmate connectedness and reported lower levels of bullying perpetration. Those with highly educated parents reported more physical and emotional complaints and fatigue, but less cyberbullying. Finally, students with a migration background reported higher levels of loneliness, bullying perpetration, cyberbullying victimization and perpetration, and lower levels of school belonging and classmate connectedness.

Notably, students attending schools with a higher socioeconomic composition reported less problematic social media use, fewer emotional complaints and less fatigue, although no differences were observed in physical complaints. Students from large schools reported less bullying perpetration than their peers in small schools.

## Discussion

More and more countries are implementing smartphone ban policies at schools. There are variations in the type of ban that schools are implementing: some schools apply smartphone restrictions to the classroom only (partial bans), while others extend the restrictions to the whole school grounds (full bans), hoping to foster student well-being and strengthen social connectedness at school. However, there is currently limited robust empirical evidence that stricter policies are more effective in achieving these intended benefits. This study examined whether different types of school smartphone ban policies are associated with adolescents’ well-being and social connectedness at school, and whether these associations vary by sex. Contrary to expectations, there were not more positive outcomes for schools with full bans compared to schools with partial bans. Moreover, in schools with a full ban boys and girls reported lower levels of student-teacher connectedness and girls reported lower levels of school belonging. It appears that stricter smartphone bans do not yield beneficial effects for students’ well-being or bullying and may even undermine students’ social connectedness at school.

Given robust evidence linking problematic social media use to poorer well-being (Sohn et al., 2019), it was hypothesized to find positive associations between stricter bans and student well-being. However, no association was found between the type of smartphone ban at school and any of the well-being or bullying outcomes. In other words, students in schools with partial or full smartphone bans reported similar levels of well-being and involvement in bullying at school and cyberbullying either as victims or perpetrators. This lack of association held for both girls and boys. These results align with previous studies that also report no significant effects of (stricter) smartphone bans on student well-being (Goodyear et al., 2025; Guldvik & Kvinnsland, 2018). Moreover, a recent meta-analysis found no significant effect of social media abstinence on well-being, suggesting that temporary restriction is not the most effective way to enhance individual well-being (Lemahieu et al., 2025). This consistency across studies indicates that stricter smartphone ban policies, restricting smartphone use on the whole school grounds and during breaks, are not having the intended beneficial effects for student’s well-being. It appears that the relationship between smartphone ban policies and well-being outcomes may be more complex than previously assumed.

One possible explanation is that problematic use, rather than general access to smartphones, is the key driver of negative outcomes (Sohn et al., 2019). While smartphones are the primary medium through which students access social media, simply restricting their use during school hours may not address the underlying behavioural patterns associated with problematic use (e.g., compulsive checking, emotional dependence, or nighttime scrolling). These behaviours often occur outside of school hours and may be difficult to change through institutional policies alone. It is also possible that students quickly adapt to restrictions by finding alternative ways to stay connected (e.g., laptops) or shifting their usage to before and after school hours, thereby minimizing the intended impact of the policy.

A second explanation for the lack of association between stricter smartphone bans and students’ well-being might lie in oversimplifying causes of the decline in adolescent mental health. Attributing complex societal issues, such as the rise in mental health problems among adolescents, to a single factor like smartphone use risks overlooking the interplay of multiple, reinforcing influences (e.g., global crises, increasing academic pressure, heightened awareness of mental health symptoms, evolving parenting styles, etc.; Stevens, 2024). While smartphone bans are often promoted as a straightforward solution for challenges like reduced academic performance and mental health, the current study suggests that such measures might be insufficient when applied in isolation. Addressing adolescent well-being effectively requires a more systemic approach that considers the broader social, educational, and digital environments in which young people live (Stevens, 2024).

A similar note can be made for the effect of stricter smartphone bans on bullying at school and cyberbullying. While educational stakeholders often express concern that smartphones facilitate cyberbullying within school settings (Toth, 2022), empirical research presents a more complex picture. The current study aligns with previous research, stating that smartphone ban policies should not be seen as a catch-all solution (Campbell et al., 2024), particularly when students still have access to other internet-connected devices. Moreover, since cyberbullying often occurs when students are not at school (Smith et al., 2008), the effectiveness of smartphone restrictions at school may be inherently limited.

Interestingly, while the type of smartphone ban was not significantly associated with problematic social media use, it was significantly related to students’ screentime in their free time (i.e., not for schoolwork). Specifically, students attending schools with a full smartphone ban were less likely to report high daily screentime (≥ 3 h) in their free time. These findings highlight the importance of distinguishing between screentime and problematic use, as they may be influenced by different factors and therefore require different intervention strategies. School policies alone may be insufficient to target problematic social media use, highlighting yet again the benefits of a more holistic approach involving digital literacy, parental involvement, and mental health support.

Additionally, the finding that students in schools with a full smartphone ban reported lower odds of high screentime in their free time contrasts with previous research that found evidence of a compensatory increase in smartphone use *after* school hours in response to daytime restrictions (Goodyear et al., 2025). This discrepancy may be explained by ambiguity in the screentime survey question used in the current study, as students might have interpreted “free time” to include breaks during the school day as well as after school. In that case, the lower screen time reported by students in full ban schools could reflect reduced opportunities to use smartphones during school hours because of the restrictions at school, instead of a decrease in use after school hours. Another possible explanation is that the impact of smartphone bans may depend on how they are implemented and perceived. Schools that enforce bans within a broader framework of digital well-being and student support may foster healthier habits, while bans perceived as punitive or overly restrictive may provoke compensatory behaviours.

While policymakers assume that stricter bans lead to increased opportunities for social interactions at school, thereby aiming to enhance students’ sense of social connectedness, results from the current study indicate the opposite. Specifically, in schools with a full ban boys and girls reported lower levels of student-teacher connectedness and girls reported lower levels of school belonging. There were no differences for classmate connectedness. Restricting smartphone use during breaks and between classes does not appear to foster greater interactions with teachers or peers, nor does it enhance students’ sense of school belonging. These results suggest that strict smartphone bans may even unintentionally harm student-teacher relationships and girls’ sense of school belonging. A potential explanation is that students perceive stricter policies as overly controlling or it might be that these policies reduce opportunities for informal interaction.

Since the national smartphone ban policy in schools was implemented in January 2024 in the Netherlands, and data collection occurred after this policy took effect, it was not possible to compare schools with full or partial bans to those without any smartphone policy. Furthermore, information on the specific smartphone ban policies that schools had in place prior to the national mandate was not collected. For some schools, the government policy may have represented only a minor adjustment to existing rules, while for others it may have constituted a substantial change. It was also not possible to assess baseline levels of well-being and social connectedness prior to the ban, making it unclear whether schools in different policy groups (full versus partial bans) differed in outcomes beforehand. To account for this, models were adjusted for key available school-level characteristics, which helps to mitigate potential confounding. It is also possible that the effects of the ban were not yet fully observable, given the relatively short time between the policy’s introduction and measurement. Furthermore, it should also be noted that the current study did not consider whether schools were implementing programs focused on digital literacy or other educational initiatives promoting responsible smartphone use. These programs may have influenced students’ attitudes and behaviours regarding phone use, potentially moderating the effects of the type of smartphone ban policy and impacting the observed outcomes. Further research should examine the short-, intermediate-, and long-term effects of partial and full smartphone bans in schools, as some effects may be negative in the short term but positive over time. Another important yet underexplored factor is the implementation style and school context (e.g., school climate, communication of rules, availability of alternative activities) that could potentially moderate the effects of smartphone bans. Comparative studies across schools with different enforcement approaches could help explain why some bans seem to be effective, while others are not. Qualitative research should explore how students and teachers perceive and respond to smartphone ban policies, and whether they view them as supportive or punitive.

Finally, the requirement for active parental consent may have introduced selection bias, particularly underrepresenting students from lower socioeconomic backgrounds, which constrains the generalizability of the findings. Although all available school-level characteristics are accounted for and multilevel analyses were used, unmeasured contextual factors (e.g., urbanization) may still have influenced the results. Moreover, the cross-sectional design of the study prevents establishing causality, underscoring the need for future research employing longitudinal or experimental designs. Given the limited and mixed empirical research evaluating smartphone ban policies, further investigation is needed to better understand how smartphone ban policies influence students’ well-being and social connectedness at school.

## Conclusion

Worldwide smartphone ban policies are gaining popularity, but their implementation varies: some schools restrict smartphone use only within the classroom, while others extend the restrictions to the entire school grounds. By extending bans to breaks between classes, policymakers aim to foster greater social interaction and improve adolescent well-being. However, there is currently no empirical evidence supporting the assumption that stricter bans achieve these intended benefits. This study investigated the effects of different types of smartphone ban policies on adolescents’ well-being and social connectedness at school. Results showed no significant differences in well-being or bullying outcomes between schools with full versus partial bans. Moreover, students from partial and full ban schools reported similar levels of problematic social media use. Importantly, students in schools with full bans reported lower levels of student-teacher connectedness, and girls reported lower levels of school belonging. Taken together, these findings suggest that stricter bans do not yield the anticipated benefits for adolescents’ well-being or bullying. On the contrary, it appears that they even undermine adolescents’ social connectedness at school. The assumption that stricter smartphone bans can serve as a potential solution to declining adolescent mental health was not supported. It seems that strict bans may be perceived as punitive or overly restrictive, potentially leading to harmful social effects. Instead of enforcing stricter smartphone ban policies, it might be worth exploring if policies embedded in a broader framework of digital well-being and student support foster healthier habits.

**Table 3:** Multilevel regression analyses for outcomes problematic social media use (linear) and screentime (logistic); for the full sample and stratified by sex

	Problematic social media use									
	Full sample			Male			Female			
*Predictors*	*β*	*CI*	*p*	*β*	*CI*	*p*		*β*	*CI*	*p*
(Intercept)	1.49	1.30–1.68	**< 0.001**	2.24	1.53–2.95	**< 0.001**		1.71	1.09–2.32	**< 0.001**
Sex - female	0.29	0.21–0.37	**< 0.001**	-	-	**-**		-	-	**-**
Age	0.05	0.01–0.08	**0.009**	0.07	0.01–0.12	**0.013**		0.02	-0.02–0.07	0.338
Educational track – havo	-0.15	-0.35–0.05	0.153	0.08	-0.22–0.38	0.601		0.10	-0.18–0.39	0.473
Educational track – vwo	0.04	-0.18–0.26	0.734	0.03	-0.14–0.20	0.730		0.31	0.15–0.46	**< 0.001**
Highest parental education - mid	-0.08	-0.25–0.10	0.393	-0.53	-0.77 – -0.28	**< 0.001**		-0.11	-0.34–0.13	0.371
Highest parental education - high	-0.13	-0.30–0.03	0.115	-0.45	-0.68 – -0.21	**< 0.001**		-0.10	-0.32–0.11	0.342
Migration background - yes	0.05	-0.05–0.14	0.342	-0.05	-0.19–0.09	0.466		0.17	0.04–0.30	**0.013**
School socio-economic composition	-0.00	-0.00 – -0.00	**0.014**	-0.00	-0.01–0.00	0.215		-0.00	-0.00–0.00	0.255
School size - large	0.04	-0.17–0.25	0.701	-0.03	-0.36–0.30	0.855		-0.15	-0.42–0.13	0.293
Smartphone ban type – full	-0.03	-0.16–0.09	0.597	-0.20	-0.43–0.02	0.074		-0.04	-0.19–0.12	0.646
**Random effects**										
σ^2^	0.60			0.60				0.61		
τ_00 school_	0.01			0.04				0.01		
ICC	0.01			0.06				0.01		
Marginal R^2^ / Conditional R^2^	0.064 / 0.076			0.064 / 0.123				0.053 / 0.063		

	Screentime									
	Full sample			Male			Female			
*Predictors*	*OR*	*CI*	*p*	*OR*	*CI*	*p*		*OR*	*CI*	*P*
(Intercept)	0.92	0.40–2.12	0.839	0.66	0.23–1.88	**0.**438		0.66	0.13–3.41	0.617
Sex - female	0.97	0.78–1.20	0.779	**-**	**-**	**-**		**-**	**-**	**-**
Age	1.14	1.05–1.24	**0.002**	1.13	1.01–1.27	**0.037**		1.17	1.04–1.33	**0.011**
Educational track – havo	0.87	0.54–1.40	0.560	1.09	0.59–2.00	0.787		1.64	0.77–3.51	0.203
Educational track – vwo	0.73	0.42–1.27	0.272	0.99	0.49–2.02	0.979		0.75	0.50–1.14	0.175
Highest parental education - mid	1.22	0.79–1.89	0.366	0.82	0.43–1.55	0.537		1.62	0.88–2.97	0.123
Highest parental education - high	1.38	0.92–2.08	0.124	1.52	0.84–2.77	0.169		1.16	0.66–2.06	0.605
Migration background - yes	1.17	0.91–1.49	0.219	1.17	0.82–1.67	0.378		1.25	0.88–1.78	0.209
School socio-economic composition	1.00	1.00–1.01	0.823	1.00	1.00–1.01	0.655		1.00	0.99–1.01	0.785
School size - large	1.06	0.66–1.71	0.797	1.10	0.59–2.02	0.769		1.10	0.52–2.33	0.806
Smartphone ban type – full	0.75	0.58–0.98	**0.038**	0.78	0.56–1.07	0.128		0.76	0.50–1.15	0.196
**Random effects**										
σ^2^	3.29			3.29				3.29		
τ_00 school_	0.01			0.00				0.05		
ICC	0.00			0.00				0.01		
Marginal R^2^ / Conditional R^2^	0.023 / 0.027			0.036 / 0.036				0.055 / 0.068		

Bold values indicate statistical significance at *p* < 0.05

**Table 4:** Multilevel linear regression analyses for all well-being outcomes (n = 1398)

	Full sample			Male			Female		
*Predictors*	*β*	*CI*	*p*	*β*	*CI*	*p*	*β*	*CI*	*p*
**Life satisfaction**									
(Intercept)	7.52	7.17 – 7.87	<0.001	7.22	5.90 – 8.55	<0.001	6.58	5.52 – 7.63	<0.001
Sex - female	-0.44	-0.59 – -0.29	<0.001						
Age	-0.05	-0.12 – 0.01	0.099	-0.05	-0.15 – 0.06	0.363	-0.08	-0.16 – 0.00	0.064
Educational track – havo	-0.17	-0.55 – 0.20	0.358	0.43	-0.15 – 1.01	0.144	0.11	-0.39 – 0.60	0.677
Educational track – vwo	-0.12	-0.53 – 0.29	0.558	0.03	-0.31 – 0.36	0.883	-0.04	-0.32 – 0.25	0.802
Highest parental education - mid	-0.05	-0.37 – 0.27	0.758	-0.16	-0.65 – 0.34	0.540	0.07	-0.36 – 0.50	0.762
Highest parental education - high	-0.02	-0.32 – 0.28	0.896	-0.09	-0.56 – 0.38	0.704	0.21	-0.20 – 0.61	0.313
Migration background - yes	0.06	-0.12 – 0.24	0.524	-0.13	-0.41 – 0.14	0.343	0.11	-0.14 – 0.35	0.393
School socio-economic composition	0.00	-0.00 – 0.01	0.262	0.00	-0.00 – 0.01	0.348	0.00	-0.00 – 0.01	0.068
School size - large	0.29	-0.10 – 0.67	0.147	0.45	-0.16 – 1.05	0.152	0.12	-0.34 – 0.58	0.604
Smartphone ban type – full	0.03	-0.21 – 0.26	0.820	-0.16	-0.55 – 0.23	0.419	0.02	-0.22 – 0.26	0.877
**Random effects**									
σ^2^	2.04			2.46			2.13		
τ_00 school_	0.03			0.10			0.00		
ICC	0.01			0.04			0.01		
Marginal R^2^ / Conditional R^2^	0.031 / 0.045			0.019 / 0.058			0.014 / 0.016		
**Loneliness**									
(Intercept)	2.59	2.28 – 2.91	<0.001	3.48	2.33 – 4.63	<0.001	2.31	1.37 – 3.24	<0.001
Sex - female	0.20	0.08 – 0.32	0.001	**-**	**-**	**-**	**-**	**-**	**-**
Age	0.01	-0.04 – 0.07	0.685	0.02	-0.07 – 0.10	0.708	0.03	-0.03 – 0.10	0.307
Educational track – havo	0.02	-0.31 – 0.36	0.898	-0.22	-0.70 – 0.26	0.362	0.12	-0.30 – 0.54	0.578
Educational track – vwo	-0.01	-0.36 – 0.35	0.972	-0.01	-0.26 – 0.24	0.952	-0.01	-0.22 – 0.19	0.902
Highest parental education - mid	-0.12	-0.37 – 0.14	0.366	-0.42	-0.79 – -0.05	0.026	-0.19	-0.50 – 0.12	0.223
Highest parental education - high	-0.13	-0.37 – 0.11	0.285	-0.61	-0.96 – -0.27	0.001	-0.21	-0.50 – 0.08	0.155
Migration background - yes	0.23	0.09 – 0.38	0.001	0.22	0.01 – 0.42	0.039	0.11	-0.07 – 0.29	0.239
School socio-economic composition	-0.00	-0.01 – 0.00	0.110	-0.00	-0.01 – 0.00	0.266	-0.00	-0.01 – 0.00	0.125
School size - large	-0.16	-0.53 – 0.21	0.392	-0.39	-0.93 – 0.15	0.161	0.14	-0.29 – 0.57	0.511
Smartphone ban type – full	-0.02	-0.28 – 0.23	0.860	-0.08	-0.47 – 0.31	0.678	0.05	-0.21 – 0.32	0.688
**Random effects**									
σ^2^	1.28			1.32			1.07		
τ_00 school_	0.06			0.14			0.04		
ICC	0.04			0.10			0.04		
Marginal R^2^ / Conditional R^2^	0.029 / 0.071			0.051 / 0.143			0.017 / 0.051		
**Physical complaints**									
(Intercept)	0.21	0.08 – 0.34	0.002	0.53	0.14 – 0.93	0.007	0.26	-0.25 – 0.78	0.314
Sex - female	0.31	0.25 – 0.37	<0.001	**-**	**-**	**-**	**-**	**-**	**-**
Age	0.02	-0.01 – 0.04	0.134	0.01	-0.02 – 0.04	0.502	0.03	-0.01 – 0.07	0.113
Educational track – havo	-0.01	-0.15 – 0.13	0.877	-0.13	-0.30 – 0.05	0.157	0.17	-0.06 – 0.41	0.150
Educational track – vwo	-0.03	-0.19 – 0.13	0.679	-0.02	-0.13 – 0.08	0.658	-0.03	-0.15 – 0.10	0.675
Highest parental education - mid	-0.21	-0.33 – -0.09	0.001	-0.20	-0.36 – -0.04	0.014	-0.22	-0.41 – -0.04	0.018
Highest parental education - high	-0.22	-0.34 – -0.11	<0.001	-0.22	-0.37 – -0.07	0.004	-0.23	-0.40 – -0.06	0.008
Migration background - yes	0.05	-0.02 – 0.11	0.199	0.06	-0.03 – 0.15	0.202	0.03	-0.07 – 0.14	0.547
School socio-economic composition	-0.00	-0.00 – 0.00	0.075	-0.00	-0.00 – 0.00	0.580	-0.00	-0.00 – 0.00	0.073
School size - large	-0.06	-0.21 – 0.08	0.392	-0.19	-0.37 – -0.01	0.040	0.09	-0.15 – 0.32	0.462
Smartphone ban type – full	-0.06	-0.15 – 0.03	0.179	-0.08	-0.19 – 0.03	0.141	-0.03	-0.16 – 0.11	0.712
**Random effects**									
σ^2^	0.32			0.25			0.38		
τ_00 school_	0.00			0.01			0.01		
ICC	0.01			0.02			0.02		
Marginal R^2^ / Conditional R^2^	0.093 / 0.102			0.045 / 0.067			0.026 / 0.044		
**Emotional complaints**									
(Intercept)	0.14	0.01 – 0.28	0.040	0.60	0.19 – 1.02	0.004	-0.07	-0.60 – 0.45	0.788
Sex - female	0.29	0.23 – 0.35	<0.001	**-**	**-**	**-**	**-**	**-**	**-**
Age	0.03	0.01 – 0.06	0.011	0.03	-0.01 – 0.06	0.104	0.04	0.01 – 0.08	0.025
Educational track – havo	-0.03	-0.18 – 0.12	0.718	-0.15	-0.34 – 0.03	0.100	0.24	0.00 – 0.48	0.048
Educational track – vwo	0.00	-0.16 – 0.17	0.964	-0.02	-0.13 – 0.10	0.772	0.07	-0.05 – 0.19	0.269
Highest parental education - mid	-0.14	-0.26 – -0.01	0.031	-0.17	-0.34 – -0.00	0.044	-0.10	-0.29 – 0.08	0.279
Highest parental education - high	-0.20	-0.32 – -0.08	0.001	-0.25	-0.41 – -0.09	0.002	-0.15	-0.32 – 0.03	0.095
Migration background - yes	0.03	-0.04 – 0.11	0.345	0.04	-0.06 – 0.13	0.428	0.02	-0.08 – 0.13	0.673
School socio-economic composition	-0.00	-0.00 – -0.00	0.009	-0.00	-0.00 – 0.00	0.205	-0.00	-0.01 – -0.00	0.021
School size - large	-0.06	-0.21 – 0.10	0.472	-0.21	-0.40 – -0.02	0.028	0.15	-0.09 – 0.38	0.228
Smartphone ban type – full	-0.04	-0.13 – 0.05	0.395	-0.07	-0.19 – 0.04	0.193	0.02	-0.12 – 0.16	0.797
**Random effects**									
σ^2^	0.34			0.29			0.39		
τ_00 school_	0.00			0.01			0.01		
ICC	0.01			0.02			0.02		
Marginal R^2^ / Conditional R^2^	0.082 / 0.092			0.050 / 0.068			0.034 / 0.054		
**Fatigue**									
(Intercept)	0.16	0.02 – 0.29	0.022	0.58	0.17 – 0.99	0.005	-0.22	-0.71 – 0.26	0.373
Sex - female	0.22	0.16 – 0.28	<0.001	**-**	**-**	**-**	**-**	**-**	**-**
Age	0.04	0.02 – 0.07	0.001	0.03	-0.00 – 0.07	0.061	0.06	0.02 – 0.10	0.001
Educational track – havo	-0.04	-0.18 – 0.11	0.609	-0.14	-0.32 – 0.05	0.144	0.26	0.04 – 0.48	0.023
Educational track – vwo	-0.01	-0.18 – 0.15	0.872	0.03	-0.09 – 0.15	0.660	0.02	-0.10 – 0.14	0.745
Highest parental education - mid	-0.13	-0.25 – -0.00	0.046	-0.18	-0.36 – -0.01	0.040	-0.08	-0.26 – 0.10	0.402
Highest parental education - high	-0.22	-0.34 – -0.10	<0.001	-0.28	-0.44 – -0.11	0.001	-0.16	-0.33 – 0.00	0.055
Migration background - yes	0.03	-0.04 – 0.10	0.441	0.03	-0.07 – 0.13	0.553	0.02	-0.08 – 0.12	0.722
School socio-economic composition	-0.00	-0.00 – -0.00	0.030	-0.00	-0.00 – 0.00	0.240	-0.00	-0.00 – -0.00	0.039
School size - large	-0.01	-0.16 – 0.14	0.920	-0.18	-0.36 – 0.01	0.058	0.21	-0.01 – 0.42	0.063
Smartphone ban type – full	-0.04	-0.12 – 0.05	0.427	-0.08	-0.18 – 0.03	0.149	0.04	-0.08 – 0.16	0.529
**Random effects**									
σ^2^	0.35			0.32			0.36		
τ_00 school_	0.00			0.00			0.00		
ICC	0.01			0.01			0.01		
Marginal R^2^ / Conditional R^2^	0.062 / 0.070			0.044 / 0.052			0.040 / 0.051		

Bold values indicate statistical significance at p < 0.05

**Table 5:** Multilevel linear regression analyses for social connectedness at school outcomes (n = 1398)

	Full sample			Male			Female		
*Predictors*	*β*	*CI*	*p*	*β*	*CI*	*p*	*β*	*CI*	*p*
**School belonging**									
(Intercept)	3.26	3.01 – 3.51	<0.001	2.77	2.06 – 3.49	<0.001	3.95	3.32 – 4.58	<0.001
Sex - female	-0.00	-0.10 – 0.09	0.938	**-**	**-**	**-**	**-**	**-**	**-**
Age^a^	0.05	0.01 – 0.10	0.017	0.06	0.00 – 0.11	0.049	0.02	-0.03 – 0.07	0.383
Educational track – havo	0.09	-0.17 – 0.36	0.489	0.06	-0.25 – 0.37	0.693	-0.37	-0.66 – -0.09	0.011
Educational track – vwo	0.15	-0.13 – 0.44	0.280	0.00	-0.18 – 0.18	0.994	0.03	-0.12 – 0.17	0.732
Highest parental education - mid	0.15	-0.06 – 0.35	0.158	0.34	0.08 – 0.60	0.010	0.24	0.02 – 0.47	0.032
Highest parental education - high	0.09	-0.11 – 0.28	0.383	0.42	0.18 – 0.67	0.001	0.21	0.00 – 0.42	0.045
Migration background - yes	-0.18	-0.29 – -0.06	0.002	-0.03	-0.18 – 0.11	0.652	-0.02	-0.15 – 0.11	0.730
School socio-economic composition	-0.00	-0.01 – 0.00	0.292	-0.00	-0.01 – 0.00	0.053	-0.00	-0.00 – 0.00	0.979
School size - large	0.18	-0.11 – 0.47	0.230	0.30	-0.04 – 0.63	0.080	-0.07	-0.36 – 0.21	0.622
Smartphone ban type – full	-0.03	-0.23 – 0.17	0.783	0.02	-0.20 – 0.24	0.851	-0.18	-0.35 – -0.01	0.036
**Random effects**									
σ^2^	0.82			0.66			0.56		
τ_00 school_	0.03			0.04			0.01		
ICC	0.04			0.05			0.02		
Marginal R^2^ / Conditional R^2^	0.039 / 0.077			0.076 / 0.124			0.049 / 0.067		
**Student-teacher connectedness**									
(Intercept)	3.98	3.77 – 4.18	<0.001	3.96	3.33 – 4.60	<0.001	4.10	3.48 – 4.73	<0.001
Sex - female	-0.21	-0.29 – -0.12	<0.001						
Age^a^	-0.02	-0.06 – 0.01	0.233	0.03	-0.03 – 0.08	0.326	-0.02	-0.07 – 0.03	0.460
Educational track – havo	0.11	-0.11 – 0.33	0.342	0.02	-0.27 – 0.30	0.906	-0.04	-0.33 – 0.25	0.789
Educational track – vwo	0.13	-0.11 – 0.37	0.289	0.03	-0.17 – 0.23	0.775	-0.01	-0.18 – 0.16	0.906
Highest parental education - mid	0.14	-0.05 – 0.32	0.146	0.41	0.11 – 0.70	0.007	0.00	-0.25 – 0.26	0.986
Highest parental education - high	-0.01	-0.18 – 0.17	0.934	0.29	0.01 – 0.57	0.039	-0.09	-0.34 – 0.15	0.439
Migration background - yes	0.09	-0.01 – 0.19	0.076	0.07	-0.10 – 0.23	0.409	-0.03	-0.18 – 0.11	0.640
School socio-economic composition	0.00	-0.00 – 0.00	0.555	-0.00	-0.00 – 0.00	0.641	-0.00	-0.00 – 0.00	0.465
School size - large	-0.16	-0.39 – 0.07	0.184	-0.16	-0.44 – 0.13	0.280	-0.04	-0.32 – 0.23	0.772
Smartphone ban type – full	-0.14	-0.29 – 0.01	0.068	-0.23	-0.38 – -0.08	0.003	-0.18	-0.33 – -0.04	0.013
**Random effects**									
σ^2^	0.66			0.89			0.75		
τ_00 school_	0.01			0.00			0.00		
ICC	0.02			0.02			0.01		
Marginal R^2^ / Conditional R^2^	0.033 / 0.053			0.031 / 0.049			0.014 / 0.016		
**Classmate connectedness**									
(Intercept)	3.45	3.20 – 3.70	<0.001	3.53	2.78 – 4.27	<0.001	4.13	3.49 – 4.77	<0.001
Sex - female	0.03	-0.07 – 0.13	0.566	-	-	-	-	-	-
Age^a^	0.05	0.00 – 0.10	0.031	0.00	-0.05 – 0.06	0.871	-0.02	-0.07 – 0.02	0.299
Educational track – havo	0.20	-0.07 – 0.47	0.153	-0.25	-0.58 – 0.07	0.123	-0.24	-0.53 – 0.06	0.116
Educational track – vwo	0.34	0.05 – 0.63	0.023	-0.09	-0.27 – 0.10	0.364	0.17	0.01 – 0.32	0.036
Highest parental education - mid	0.06	-0.16 – 0.28	0.591	0.50	0.22 – 0.77	<0.001	0.13	-0.11 – 0.36	0.285
Highest parental education - high	-0.09	-0.29 – 0.12	0.392	0.41	0.15 – 0.66	0.002	-0.03	-0.25 – 0.19	0.798
Migration background - yes	-0.24	-0.36 – -0.12	<0.001	0.14	-0.01 – 0.29	0.073	-0.03	-0.17 – 0.11	0.663
School socio-economic composition	-0.00	-0.00 – 0.00	0.859	0.00	-0.00 – 0.00	0.698	0.00	-0.00 – 0.00	0.435
School size - large	0.07	-0.22 – 0.36	0.646	-0.02	-0.36 – 0.33	0.918	-0.09	-0.38 – 0.20	0.544
Smartphone ban type – full	-0.08	-0.27 – 0.12	0.426	0.02	-0.21 – 0.24	0.878	-0.10	-0.27 – 0.07	0.248
**Random effects**									
σ^2^	0.92			0.73			0.62		
τ_00 school_	0.03			0.04			0.01		
ICC	0.03			0.05			0.01		
Marginal R^2^ / Conditional R^2^	0.039 / 0.068			0.034 / 0.080			0.021 / 0.035		

Bold values indicate statistical significance at p < 0.05

**Table 6:** Multilevel linear regression analyses for bullying outcomes (n = 1398)

	Full sample			Male			Female		
*Predictors*	*β*	*CI*	*p*	*β*	*CI*	*p*	*β*	*CI*	*p*
**Bullying at school victimization**									
(Intercept)	1.30	1.15 – 1.45	<0.001	2.29	1.52 – 3.07	<0.001	1.56	0.86 – 2.26	<0.001
Sex - female	-0.04	-0.11 – 0.02	0.217	-	-	-	-	-	-
Age	-0.01	-0.04 – 0.01	0.334	-0.07	-0.13 – -0.00	0.044	-0.04	-0.09 – 0.01	0.166
Educational track – havo	-0.04	-0.20 – 0.13	0.669	-0.06	-0.41 – 0.28	0.722	-0.01	-0.32 – 0.31	0.973
Educational track – vwo	-0.07	-0.25 – 0.11	0.441	-0.05	-0.26 – 0.16	0.649	-0.08	-0.24 – 0.08	0.305
Highest parental education - mid	-0.07	-0.21 – 0.07	0.336	-0.33	-0.64 – -0.01	0.042	-0.20	-0.44 – 0.03	0.089
Highest parental education - high	0.02	-0.12 – 0.15	0.785	-0.26	-0.56 – 0.04	0.087	-0.18	-0.40 – 0.04	0.111
Migration background - yes	-0.04	-0.12 – 0.03	0.273	0.06	-0.12 – 0.23	0.540	0.02	-0.12 – 0.16	0.791
School socio-economic composition	-0.00	-0.00 – 0.00	0.118	-0.00	-0.00 – 0.00	0.778	-0.00	-0.00 – 0.00	0.987
School size - large	-0.07	-0.24 – 0.09	0.392	-0.31	-0.66 – 0.05	0.092	-0.08	-0.41 – 0.24	0.612
Smartphone ban type – full	0.03	-0.07 – 0.13	0.533	-0.05	-0.26 – 0.16	0.643	0.05	-0.15 – 0.25	0.613
**Random effects**									
σ^2^	0.40			1.00			0.63		
τ_00 school_	0.00			0.02			0.02		
ICC	0.01			0.02			0.03		
Marginal R^2^ / Conditional R^2^	0.021 / 0.032			0.038 / 0.058			0.017 / 0.047		
**Bullying at school perpetration**									
(Intercept)	2.06	1.72 – 2.40	<0.001	2.63	1.26 – 3.99	<0.001	1.94	1.13 – 2.75	<0.001
Sex - female	-0.37	-0.50 – -0.25	<0.001	-	-	-	-	-	-
Age	-0.03	-0.09 – 0.03	0.294	0.02	-0.07 – 0.12	0.665	-0.05	-0.11 – 0.00	0.060
Educational track – havo	0.20	-0.15 – 0.56	0.259	0.03	-0.52 – 0.59	0.903	-0.00	-0.36 – 0.36	0.995
Educational track – vwo	0.14	-0.23 – 0.51	0.461	-0.08	-0.37 – 0.20	0.566	-0.02	-0.19 – 0.15	0.835
Highest parental education - mid	-0.36	-0.62 – -0.09	0.008	-0.58	-1.00 – -0.16	0.007	-0.66	-0.91 – -0.40	<0.001
Highest parental education - high	-0.23	-0.48 – 0.02	0.066	-0.48	-0.87 – -0.09	0.017	-0.57	-0.81 – -0.34	<0.001
Migration background - yes	0.17	0.02 – 0.31	0.024	0.13	-0.11 – 0.36	0.292	0.07	-0.08 – 0.22	0.335
School socio-economic composition	0.00	-0.00 – 0.01	0.418	0.00	-0.01 – 0.01	0.684	0.00	-0.00 – 0.01	0.264
School size - large	-0.43	-0.84 – -0.03	0.036	-0.42	-1.06 – 0.22	0.202	-0.23	-0.60 – 0.15	0.236
Smartphone ban type – full	0.05	-0.25 – 0.34	0.750	0.05	-0.42 – 0.53	0.825	0.10	-0.14 – 0.34	0.424
**Random effects**									
σ^2^	1.34			1.68			0.72		
τ_00 school_	0.08			0.23			0.04		
ICC	0.06			0.12			0.05		
Marginal R^2^ / Conditional R^2^	0.049 / 0.103			0.045 / 0.159			0.069 / 0.116		
**Cyberbullying victimization**									
(Intercept)	1.91	1.57 – 2.25	<0.001	0.69	0.00 – 1.37	0.050	0.89	0.39 – 1.39	<0.001
Sex - female	-0.20	-0.29 – -0.10	<0.001	-	-	-	-	-	-
Age^a^	-0.07	-0.12 – -0.02	0.005	-0.07	-0.12 – -0.01	0.021	-0.03	-0.06 – 0.01	0.190
Educational track – havo	-0.00	-0.31 – 0.30	0.982	0.17	-0.13 – 0.48	0.262	0.12	-0.11 – 0.36	0.295
Educational track – vwo	-0.02	-0.33 – 0.30	0.912	0.08	-0.10 – 0.27	0.389	-0.03	-0.16 – 0.10	0.600
Highest parental education - mid	-0.30	-0.51 – -0.10	0.004	0.05	-0.22 – 0.32	0.713	-0.11	-0.31 – 0.08	0.261
Highest parental education - high	-0.25	-0.45 – -0.06	0.012	0.07	-0.18 – 0.33	0.581	-0.06	-0.25 – 0.12	0.497
Migration background - yes	0.12	0.00 – 0.24	0.043	0.10	-0.05 – 0.25	0.193	0.03	-0.08 – 0.15	0.545
School socio-economic composition	0.00	-0.00 – 0.01	0.744	-0.00	-0.00 – 0.00	0.707	-0.00	-0.00 – 0.00	0.843
School size - large	-0.40	-0.80 – 0.01	0.057	0.11	-0.20 – 0.43	0.475	0.06	-0.16 – 0.28	0.605
Smartphone ban type – full	0.04	-0.30 – 0.37	0.835	0.06	-0.14 – 0.25	0.550	0.12	-0.00 – 0.24	0.054
**Random effects**									
σ^2^	0.84			0.74			0.44		
τ_00 school_	0.13			0.02			0.00		
ICC	0.13			0.03			0.00		
Marginal R^2^ / Conditional R^2^	0.066 / 0.189			0.019 / 0.046			0.016 / 0.020		
**Cyberbullying perpetration**									
(Intercept)	1.98	1.68 – 2.29	<0.001	1.51	0.77 – 2.26	<0.001	1.11	0.52 – 1.69	<0.001
Sex - female	-0.31	-0.41 – -0.20	<0.001	-	-	-	-	-	-
Age	-0.04	-0.09 – 0.01	0.104	0.06	-0.00 – 0.12	0.052	-0.01	-0.05 – 0.03	0.621
Educational track – havo	-0.06	-0.37 – 0.26	0.727	0.16	-0.17 – 0.48	0.347	0.07	-0.19 – 0.32	0.603
Educational track – vwo	-0.11	-0.43 – 0.22	0.514	-0.05	-0.24 – 0.14	0.618	-0.03	-0.15 – 0.09	0.608
Highest parental education - mid	-0.44	-0.66 – -0.21	<0.001	-0.21	-0.49 – 0.07	0.149	-0.36	-0.54 – -0.19	<0.001
Highest parental education - high	-0.31	-0.52 – -0.10	0.005	-0.15	-0.42 – 0.11	0.253	-0.31	-0.47 – -0.14	<0.001
Migration background - yes	0.15	0.02 – 0.28	0.019	-0.13	-0.29 – 0.03	0.100	0.05	-0.05 – 0.16	0.302
School socio-economic composition	0.00	-0.00 – 0.01	0.363	-0.00	-0.00 – 0.00	0.982	0.00	-0.00 – 0.00	0.226
School size - large	-0.17	-0.53 – 0.19	0.362	0.09	-0.25 – 0.43	0.600	-0.01	-0.28 – 0.27	0.960
Smartphone ban type – full	0.07	-0.20 – 0.34	0.609	-0.06	-0.28 – 0.16	0.569	0.15	-0.04 – 0.33	0.120
**Random effects**									
σ^2^	1.01			0.78			0.34		
τ_00 school_	0.07			0.03			0.03		
ICC	0.07			0.04			0.07		
Marginal R^2^ / Conditional R^2^	0.053 / 0.115			0.019 / 0.057			0.051 / 0.120		

Bold values indicate statistical significance at p < 0.05

## Supplementary Information

Below is the link to the electronic supplementary material.

Supplementary Material 1

## Data Availability

The data that support the findings of this study can be made available by the authors upon reasonable request, under strict conditions. Data from the Netherlands Cohort Study on Education (NCO) regarding socioeconomic status and migration background were obtained from the National Educational Research Organisation’s (NRO). Under certain conditions, these microdata are accessible for statistical and scientific research. For further information: microdata@cbs.nl.
