# Supplementary material for: Disconnect To Reconnect: How Variations between Types of Smartphone Bans Influence Students’ Well-being and Social Connectedness in Dutch Secondary Education
Source: J Youth Adolesc. 2026 Jan 7;55(3):551–69. doi: 10.1007/s10964-025-02313-6 (PMC12966246; doi:10.1007/s10964-025-02313-6)
Supplement: Supplementary file 1 — Supplementary Material 1 [file 10964_2025_2313_MOESM1_ESM.docx]

**Disconnect to Reconnect? How Variations Between Types of Smartphone Bans Influence Students' Well-being and Social Connectedness in Dutch Secondary Education**

Elien Vanluydt, Regina van den Eijnden, Lisanne Vonk, Polina Putrik, Thérèse van Amelsvoort, Philippe Delespaul, Mark Levels, and Tim Huijts

**Appendix A**

**Supplementary Table 1.** Overview of the survey questions relevant for the present study.

| **Loneliness** | |
| --- | --- |
| How many times did you feel lonely in the last 12 months? | Never – Almost never – Sometimes – Often - Always |
| **General life satisfaction** | |
| How do you feel about your life? Below you can see a ladder from 10 to 1 with 10 representing the best life you can imagine and 1 representing the worst life you can imagine. At which spot on the ladder is your life situated in general? Choose the number that corresponds best with how you feel about your life right now. | 1 – 2 – 3 – 4 – 5 – 6 – 7 – 8 – 9 – 10 |
| **Psychosomatic complaints** | |
| In the last six months how many times did you experience …   - Having a headache - Having stomachache - Having back ache - Feeling unhappy - Being irritable - Feeling nervous - Having difficulty falling asleep - Sleeping badly - Feeling dizzy - Feeling nauseous - Having difficulty concentrating - Having daytime fatigue | Almost every day – More than once a week – Almost every week – Almost every month – Hardly ever or never |
| **School belonging** |  |
| - I like going to school - I feel safe at school - I feel excluded at school - I make friends easily at school - I feel home at school - I feel lonely at school - Other students seem to like me - I don’t feel at ease at school | Completely agree – Agree – Neutral – Disagree – Completely disagree |
| **Student-teacher connectedness** | |
| - My teacher accepts me the way I am - I feel like my teachers care about me - I have a lot of trust in my teachers | Completely agree – Agree – Neutral – Disagree – Completely disagree |
| **Classmate Connectedness** | |
| - My classmates like being with each other - Most of my classmates are friendly and helpful - My classmates accept me as I am | Completely agree – Agree – Neutral – Disagree – Completely disagree |
| **Screentime** |  |
| How many hours per day do you spend behind the computer, tablet, smartphone, or television in your free time (so not for school)?” | (Almost) never – One hour per day – Two hours per day – three hours per day – Four hours per day |
| **Problematic Social Media Use** | |
| - How many times did you find it hard to quit using social media? - How many times did others (e.g., parents or friends) tell you that you should spend less time on social media? - How many times did you chose spending time on social media over spending time in-person with others (e.g., friends or parents)? - How many times did you feel restless, stressed, or irritated while using social media? - How many times did you feel restless, stressed, or irritated when you did not have access to social media? - How many times were you on social media while you were supposed to do other things (e.g., homework)? - How many times did you use social media because you were feeling bad? - How many times did you use social media before going to sleep? | Never – almost never – sometimes – often – very often |
| **Bullying** | |
| - How many times in the last few months were you bullied at school? - How many times in the last few months did you participate in bullying someone else at school? | Never – 1 or 2 times – 2 or 3 times – once a week – a couple times a week |
| **Cyberbullying** |  |
| - How many times in the last few months were you bullied online? - How many times in the last few months did you participate in bullying someone else online? | Never – 1 or 2 times – 2 or 3 times – once a week – a couple times a week |

**Appendix B**

**Supplementary Table 2**. Results for three-factor CFA solution of psychosomatic complaints: standardized factor loadings and error variances

| **Factor** | **Item** | **Standardized Loadings** | **Error variances** |
| --- | --- | --- | --- |
| Physical complaints | Headache | 0.814 | 0.338 |
|  | Stomachache | 0.765 | 0.415 |
|  | Backache | 0.599 | 0.641 |
|  | Dizzy | 0.821 | 0.326 |
|  | Nauseous | 0.834 | 0.304 |
| Emotional complaints | Nervous | 0.816 | 0.334 |
|  | Concentration | 0.583 | 0.661 |
|  | Unhappy | 0.780 | 0.392 |
|  | Bad mood | 0.801 | 0.359 |
| Fatigue | Falling asleep | 0.829 | 0.313 |
|  | Sleeping bad | 0.934 | 0.127 |
|  | Tired | 0.833 | 0.307 |

**Appendix C**

**Supplementary Table 3.** An overview of missing values.

| **Variable** | **Total (n = 1398)** | **Boys (n = 678)** | **Girls (n = 687)** |
| --- | --- | --- | --- |
| Sex | 33 (2.4%) | - | - |
| Age | 0 (0.0%) | 0 (0.0%) | 0 (0.0%) |
| Grade | 0 (0.0%) | 0 (0.0%) | 0 (0.0%) |
| Educational track | 0 (0.0%) | 0 (0.0%) | 0 (0.0%) |
| Parental educational attainment | 430 (30.8%) | 210 (31.0%) | 195 (28.4%) |
| Migration background | 319 (22.8%) | 172 (25.4%) | 123 (17.9%) |
| School socio-economic composition | 0 (0.0%) | 0 (0.0%) | 0 (0.0%) |
| School size | 0 (0.0%) | 0 (0.0%) | 0 (0.0%) |
| Urbanization | 0 (0.0%) | 0 (0.0%) | 0 (0.0%) |
| Denomination | 0 (0.0%) | 0 (0.0%) | 0 (0.0%) |
| Smartphone ban type | 0 (0.0%) | 0 (0.0%) | 0 (0.0%) |
| Life satisfaction | 118 (8.4%) | 67 (9.9%) | 29 (4.2%) |
| Loneliness | 143 (10.2%) | 82 (12.1%) | 40 (5.8%) |
| Physical complaint – headache | 123 (8.8%) | 70 (10.3%) | 32 (4.7%) |
| Physical complaint – backache | 122 (8.7%) | 69 (10.2%) | 31 (4.5%) |
| Physical complaint – stomachache | 122 (8.7%) | 69 (10.2%) | 32 (4.7%) |
| Physical complaint – nauseous | 132 (9.4%) | 73 (10.8%) | 37 (5.2%) |
| Physical complaint – dizzy | 134 (9.6%) | 76 (11.2%) | 36 (5.2%) |
| Emotional complaints – bad mood | 127 (9.1%) | 72 (10.6%) | 33 (4.8%) |
| Emotional complaint – unhappy | 145 (10.4%) | 77 (11.4%) | 45 (6.6%) |
| Emotional complaint – nervous | 128 (9.2%) | 73 (10.8%) | 33 (4.8%) |
| Emotional complaint – difficulty concentrating | 129 (9.2%) | 74 (10.9%) | 32 (4.7%) |
| Fatigue – difficulty falling asleep | 131 (9.4%) | 72 (10.6%) | 37 (5.2%) |
| Fatigue – sleeping bad | 134 (9.6%) | 75 (11.1%) | 37 (5.2%) |
| Fatigue – being tired | 127 (9.1%) | 74 (10.9%) | 31 (4.5%) |
| Problematic social media use | 98 (7.0%) | 54 (8.0%) | 21 (3.1%) |
| Screentime | 93 (6.7%) | 46 (6.8%) | 25 (3.6%) |
| School belonging | 181 (12.9%) | 100 (14.7%) | 59 (8.6%) |
| Student-teacher connectedness | 206 (14.7%) | 116 (17.1%) | 66 (9.6%) |
| Classmate connectedness | 207 (14.8%) | 115 (17.0%) | 68 (9.9%) |
| Bullying – victim | 154 (11.0%) | 84 (12.4%) | 49 (7.1%) |
| Bullying – perpetrator | 158 (11.3%) | 91 (13.4%) | 45 (6.6%) |
| Cyberbullying – victim | 151 (10.8%) | 89 (13.1%) | 41 (6.0%) |
| Cyberbullying - perpetrator | 145 (10.4%) | 84 (12.4%) | 40 (5.8%) |

Note: Missing values also include the answer category ‘I’d rather not say’.

**Appendix D**

**Supplementary Table 4.** Linear multilevel regression analyses for problematic social media use

|  | **Model 1** | | | **Model 2** | | | **Model 3** | | |
| --- | --- | --- | --- | --- | --- | --- | --- | --- | --- |
| *Predictors* | *β* | *CI* | *p* | *β* | *CI* | *p* | *β* | *CI* | *p* |
| (Intercept) | 1.50 | 1.39 – 1.62 | **<0.001** | 1.47 | 1.27 – 1.66 | **<0.001** | 1.49 | 1.30 – 1.68 | **<0.001** |
| Sex - female |  |  |  | 0.29 | 0.20 – 0.37 | **<0.001** | 0.29 | 0.21 – 0.37 | **<0.001** |
| Age |  |  |  | 0.05 | 0.01 – 0.08 | **0.012** | 0.05 | 0.01 – 0.08 | **0.009** |
| Educational track – havo |  |  |  | -0.11 | -0.25 – 0.04 | 0.138 | -0.15 | -0.35 – 0.05 | 0.153 |
| Educational track – vwo |  |  |  | 0.10 | -0.04 – 0.24 | 0.177 | 0.04 | -0.18 – 0.26 | 0.734 |
| Highest parental education - mid |  |  |  | -0.07 | -0.25 – 0.10 | 0.417 | -0.08 | -0.25 – 0.10 | 0.393 |
| Highest parental education - high |  |  |  | -0.11 | -0.28 – 0.05 | 0.178 | -0.13 | -0.30 – 0.03 | 0.115 |
| Migration background - yes |  |  |  | 0.04 | -0.05 – 0.14 | 0.394 | 0.05 | -0.05 – 0.14 | 0.342 |
| School socio-economic composition |  |  |  |  |  |  | -0.00 | -0.00 – -0.00 | **0.014** |
| School size - large |  |  |  |  |  |  | 0.04 | -0.17 – 0.25 | 0.701 |
| Smartphone ban type – full | 0.02 | -0.13 – 0.16 | 0.835 | -0.02 | -0.16 – 0.11 | 0.764 | -0.03 | -0.16 – 0.09 | 0.597 |
| **Random effects** |  |  |  |  |  |  |  |  |  |
| σ^2^ | 0.64 |  |  | 0.61 |  |  | 0.60 |  |  |
| τ_00_ _school_ | 0.02 |  |  | 0.01 |  |  | 0.01 |  |  |
| ICC | 0.02 |  |  | 0.02 |  |  | 0.01 |  |  |
| Marginal R^2^ / Conditional R^2^ | 0.000 / 0.024 |  |  | 0.055 / 0.072 |  |  | 0.064 / 0.076 |  |  |

**Note.** Bold values indicate statistical significance at p < 0.05.

**Supplementary Table 5.** Logistic multilevel regression analyses for screentime

|  | **Model 1** | | | **Model 2** | | | **Model 3** | | |
| --- | --- | --- | --- | --- | --- | --- | --- | --- | --- |
| *Predictors* | *Odds Ratio* | *CI* | *p* | *Odds Ratio* | *CI* | *p* | *Odds Ratio* | *CI* | *p* |
| (Intercept) | 1.45 | 0.90 – 2.32 | 0.128 | 0.99 | 0.49 – 2.00 | 0.979 | 0.92 | 0.40 – 2.12 | 0.839 |
| Sex - female |  |  |  | 0.97 | 0.78 – 1.20 | 0.778 | 0.97 | 0.78 – 1.20 | 0.779 |
| Age |  |  |  | 1.14 | 1.05 – 1.24 | **0.002** | 1.14 | 1.05 – 1.24 | **0.002** |
| Educational track – havo |  |  |  | 0.91 | 0.66 – 1.26 | 0.566 | 0.87 | 0.54 – 1.40 | 0.560 |
| Educational track – vwo |  |  |  | 0.77 | 0.56 – 1.06 | 0.114 | 0.73 | 0.42 – 1.27 | 0.272 |
| Highest parental education - mid |  |  |  | 1.22 | 0.79 – 1.88 | 0.373 | 1.22 | 0.79 – 1.89 | 0.366 |
| Highest parental education - high |  |  |  | 1.37 | 0.91 – 2.06 | 0.131 | 1.38 | 0.92 – 2.08 | 0.124 |
| Migration background - yes |  |  |  | 1.17 | 0.91 – 1.49 | 0.212 | 1.17 | 0.91 – 1.49 | 0.219 |
| School socio-economic composition |  |  |  |  |  |  | 1.00 | 1.00 – 1.01 | 0.823 |
| School size - large |  |  |  |  |  |  | 1.06 | 0.66 – 1.71 | 0.797 |
| Smartphone ban type – full | 0.70 | 0.53 – 0.93 | **0.014** | 0.75 | 0.58 – 0.98 | **0.032** | 0.75 | 0.58 – 0.98 | **0.038** |
| **Random effects** |  |  |  |  |  |  |  |  |  |
| σ^2^ | 3.29 |  |  | 3.29 |  |  | 3.29 |  |  |
| τ_00_ _school_ | 0.03 |  |  | 0.01 |  |  | 0.01 |  |  |
| ICC | 0.01 |  |  | 0.00 |  |  | 0.00 |  |  |
| Marginal R^2^ / Conditional R^2^ | 0.009 / 0.017 |  |  | 0.023 / 0.027 |  |  | 0.023 / 0.027 |  |  |

**Note.** Bold values indicate statistical significance at p < 0.05.

**Supplementary Table 6.** Linear multilevel regression analyses for life satisfaction

|  | **Model 1** | | | **Model 2** | | | **Model 3** | | |
| --- | --- | --- | --- | --- | --- | --- | --- | --- | --- |
| *Predictors* | *β* | *CI* | *p* | *β* | *CI* | *P* | *β* | *CI* | *p* |
| (Intercept) | 7.39 | 7.17 – 7.61 | **<0.001** | 7.55 | 7.19 – 7.92 | **<0.001** | 7.52 | 7.17 – 7.87 | **<0.001** |
| Sex - female |  |  |  | -0.44 | -0.59 – -0.29 | **<0.001** | -0.44 | -0.59 – -0.29 | **<0.001** |
| Age |  |  |  | -0.05 | -0.12 – 0.02 | 0.161 | -0.05 | -0.12 – 0.01 | 0.099 |
| Educational track – havo |  |  |  | 0.02 | -0.25 – 0.30 | 0.871 | -0.17 | -0.55 – 0.20 | 0.358 |
| Educational track – vwo |  |  |  | 0.08 | -0.20 – 0.35 | 0.585 | -0.12 | -0.53 – 0.29 | 0.558 |
| Highest parental education - mid |  |  |  | -0.05 | -0.37 – 0.27 | 0.755 | -0.05 | -0.37 – 0.27 | 0.758 |
| Highest parental education - high |  |  |  | -0.02 | -0.33 – 0.28 | 0.872 | -0.02 | -0.32 – 0.28 | 0.896 |
| Migration background - yes |  |  |  | 0.06 | -0.11 – 0.24 | 0.486 | 0.06 | -0.12 – 0.24 | 0.524 |
| School socio-economic composition |  |  |  |  |  |  | 0.00 | -0.00 – 0.01 | 0.262 |
| School size - large |  |  |  |  |  |  | 0.29 | -0.10 – 0.67 | 0.147 |
| Smartphone ban type – full | 0.02 | -0.25 – 0.29 | 0.899 | 0.03 | -0.23 – 0.30 | 0.803 | 0.03 | -0.21 – 0.26 | 0.820 |
| **Random effects** |  |  |  |  |  |  |  |  |  |
| σ^2^ | 2.09 |  |  | 2.04 |  |  | 2.04 |  |  |
| τ_00_ _school_ | 0.06 |  |  | 0.05 |  |  | 0.03 |  |  |
| ICC | 0.03 |  |  | 0.02 |  |  | 0.01 |  |  |
| Marginal R^2^ / Conditional R^2^ | 0.000 / 0.027 |  |  | 0.025 / 0.048 |  |  | 0.031 / 0.045 |  |  |

**Note.** Bold values indicate statistical significance at p < 0.05.

**Supplementary Table 7.** Linear multilevel regression analyses for loneliness

|  | **Model 1** | | | **Model 2** | | | **Model 3** | | |
| --- | --- | --- | --- | --- | --- | --- | --- | --- | --- |
| *Predictors* | *β* | *CI* | *p* | *β* | *CI* | *p* | *β* | *CI* | *p* |
| (Intercept) | 2.56 | 2.36 – 2.76 | **<0.001** | 2.55 | 2.23 – 2.87 | **<0.001** | 2.59 | 2.28 – 2.91 | **<0.001** |
| Sex - female |  |  |  | 0.20 | 0.08 – 0.32 | **0.001** | 0.20 | 0.08 – 0.32 | **0.001** |
| Age |  |  |  | 0.01 | -0.04 – 0.07 | 0.666 | 0.01 | -0.04 – 0.07 | 0.685 |
| Educational track – havo |  |  |  | -0.05 | -0.30 – 0.19 | 0.667 | 0.02 | -0.31 – 0.36 | 0.898 |
| Educational track – vwo |  |  |  | -0.08 | -0.33 – 0.16 | 0.511 | -0.01 | -0.36 – 0.35 | 0.972 |
| Highest parental education - mid |  |  |  | -0.12 | -0.37 – 0.14 | 0.372 | -0.12 | -0.37 – 0.14 | 0.366 |
| Highest parental education - high |  |  |  | -0.12 | -0.36 – 0.12 | 0.319 | -0.13 | -0.37 – 0.11 | 0.285 |
| Migration background - yes |  |  |  | 0.23 | 0.09 – 0.37 | **0.002** | 0.23 | 0.09 – 0.38 | **0.001** |
| School socio-economic composition |  |  |  |  |  |  | -0.00 | -0.01 – 0.00 | 0.110 |
| School size - large |  |  |  |  |  |  | -0.16 | -0.53 – 0.21 | 0.392 |
| Smartphone ban type – full | -0.02 | -0.27 – 0.23 | 0.858 | -0.01 | -0.28 – 0.25 | 0.914 | -0.02 | -0.28 – 0.23 | 0.860 |
| **Random effects** |  |  |  |  |  |  |  |  |  |
| σ^2^ | 1.30 |  |  | 1.28 |  |  | 1.28 |  |  |
| τ_00_ _school_ | 0.06 |  |  | 0.06 |  |  | 0.06 |  |  |
| ICC | 0.04 |  |  | 0.05 |  |  | 0.04 |  |  |
| Marginal R^2^ / Conditional R^2^ | 0.000 / 0.043 |  |  | 0.019 / 0.066 |  |  | 0.029 / 0.071 |  |  |

**Note.** Bold values indicate statistical significance at p < 0.05.

**Supplementary Table 8.** Linear multilevel regression analyses for physical complaints

|  | **Model 1** | | | **Model 2** | | | **Model 3** | | |
| --- | --- | --- | --- | --- | --- | --- | --- | --- | --- |
| *Predictors* | *β* | *CI* | *p* | *β* | *CI* | *p* | *β* | *CI* | *p* |
| (Intercept) | 0.11 | 0.03 – 0.19 | **0.005** | 0.20 | 0.06 – 0.33 | **0.005** | 0.21 | 0.08 – 0.34 | **0.002** |
| Sex - female |  |  |  | 0.31 | 0.25 – 0.37 | **<0.001** | 0.31 | 0.25 – 0.37 | **<0.001** |
| Age |  |  |  | 0.02 | -0.01 – 0.04 | 0.170 | 0.02 | -0.01 – 0.04 | 0.134 |
| Educational track – havo |  |  |  | -0.06 | -0.16 – 0.05 | 0.301 | -0.01 | -0.15 – 0.13 | 0.877 |
| Educational track – vwo |  |  |  | -0.08 | -0.18 – 0.03 | 0.148 | -0.03 | -0.19 – 0.13 | 0.679 |
| Highest parental education - mid |  |  |  | -0.21 | -0.33 – -0.09 | **0.001** | -0.21 | -0.33 – -0.09 | **0.001** |
| Highest parental education - high |  |  |  | -0.22 | -0.33 – -0.10 | **<0.001** | -0.22 | -0.34 – -0.11 | **<0.001** |
| Migration background - yes |  |  |  | 0.04 | -0.03 – 0.11 | 0.223 | 0.05 | -0.02 – 0.11 | 0.199 |
| School socio-economic composition |  |  |  |  |  |  | -0.00 | -0.00 – 0.00 | 0.075 |
| School size - large |  |  |  |  |  |  | -0.06 | -0.21 – 0.08 | 0.392 |
| Smartphone ban type – full | -0.05 | -0.15 – 0.05 | 0.335 | -0.05 | -0.15 – 0.05 | 0.304 | -0.06 | -0.15 – 0.03 | 0.179 |
| **Random effects** |  |  |  |  |  |  |  |  |  |
| σ^2^ | 0.35 |  |  | 0.32 |  |  |  | 0.32 |  |
| τ_00_ _school_ | 0.01 |  |  | 0.01 |  |  |  | 0.00 |  |
| ICC | 0.02 |  |  | 0.02 |  |  |  | 0.01 |  |
| Marginal R^2^ / Conditional R^2^ | 0.002 / 0.019 |  |  | 0.089 / 0.108 |  |  |  | 0.093 / 0.102 |  |

**Note.** Bold values indicate statistical significance at p < 0.05.

**Supplementary Table 9.** Linear multilevel regression analyses for emotional complaints

|  | **Model 1** | | | **Model 2** | | | **Model 3** | | |
| --- | --- | --- | --- | --- | --- | --- | --- | --- | --- |
| *Predictors* | *β* | *CI* | *p* | *β* | *CI* | *p* | *β* | *CI* | *p* |
| (Intercept) | 0.07 | -0.02 – 0.16 | 0.125 | 0.12 | -0.02 – 0.27 | 0.100 | 0.14 | 0.01 – 0.28 | **0.040** |
| Sex - female |  |  |  | 0.29 | 0.23 – 0.35 | **<0.001** | 0.29 | 0.23 – 0.35 | **<0.001** |
| Age |  |  |  | 0.03 | 0.00 – 0.06 | **0.026** | 0.03 | 0.01 – 0.06 | **0.011** |
| Educational track – havo |  |  |  | -0.07 | -0.18 – 0.05 | 0.267 | -0.03 | -0.18 – 0.12 | 0.718 |
| Educational track – vwo |  |  |  | -0.03 | -0.15 – 0.09 | 0.616 | 0.00 | -0.16 – 0.17 | 0.964 |
| Highest parental education - mid |  |  |  | -0.13 | -0.26 – -0.00 | **0.042** | -0.14 | -0.26 – -0.01 | **0.031** |
| Highest parental education - high |  |  |  | -0.19 | -0.31 – -0.07 | **0.002** | -0.20 | -0.32 – -0.08 | **0.001** |
| Migration background - yes |  |  |  | 0.03 | -0.04 – 0.10 | 0.385 | 0.03 | -0.04 – 0.11 | 0.345 |
| School socio-economic composition |  |  |  |  |  |  | -0.00 | -0.00 – -0.00 | **0.009** |
| School size - large |  |  |  |  |  |  | -0.06 | -0.21 – 0.10 | 0.472 |
| Smartphone ban type – full | -0.02 | -0.13 – 0.10 | 0.760 | -0.03 | -0.14 – 0.08 | 0.603 | -0.04 | -0.13 – 0.05 | 0.395 |
| **Random effects** |  |  |  |  |  |  |  |  |  |
| σ^2^ | 0.37 |  |  | 0.34 |  |  | 0.34 |  |  |
| τ_00_ _school_ | 0.01 |  |  | 0.01 |  |  | 0.00 |  |  |
| ICC | 0.03 |  |  | 0.03 |  |  | 0.01 |  |  |
| Marginal R^2^ / Conditional R^2^ | 0.000 / 0.027 |  |  | 0.074 / 0.099 |  |  | 0.082 / 0.092 |  |  |

**Note.** Bold values indicate statistical significance at p < 0.05.

**Supplementary Table 10.** Linear multilevel regression analyses for fatigue

|  | **Model 1** | | | **Model 2** | | | **Model 3** | | |
| --- | --- | --- | --- | --- | --- | --- | --- | --- | --- |
| *Predictors* | *β* | *CI* | *p* | *β* | *CI* | *p* | *β* | *CI* | *p* |
| (Intercept) | 0.06 | -0.03 – 0.14 | 0.187 | 0.14 | -0.00 – 0.28 | 0.051 | 0.16 | 0.02 – 0.29 | **0.022** |
| Sex - female |  |  |  | 0.22 | 0.16 – 0.28 | **<0.001** | 0.22 | 0.16 – 0.28 | **<0.001** |
| Age |  |  |  | 0.04 | 0.02 – 0.07 | **0.002** | 0.04 | 0.02 – 0.07 | **0.001** |
| Educational track – havo |  |  |  | -0.04 | -0.15 – 0.07 | 0.428 | -0.04 | -0.18 – 0.11 | 0.609 |
| Educational track – vwo |  |  |  | -0.01 | -0.12 – 0.10 | 0.852 | -0.01 | -0.18 – 0.15 | 0.872 |
| Highest parental education - mid |  |  |  | -0.12 | -0.25 – 0.01 | 0.060 | -0.13 | -0.25 – -0.00 | **0.046** |
| Highest parental education - high |  |  |  | -0.21 | -0.33 – -0.09 | **0.001** | -0.22 | -0.34 – -0.10 | **<0.001** |
| Migration background - yes |  |  |  | 0.03 | -0.05 – 0.10 | 0.488 | 0.03 | -0.04 – 0.10 | 0.441 |
| School socio-economic composition |  |  |  |  |  |  | -0.00 | -0.00 – -0.00 | **0.030** |
| School size - large |  |  |  |  |  |  | -0.01 | -0.16 – 0.14 | 0.920 |
| Smartphone ban type – full | -0.02 | -0.13 – 0.09 | 0.698 | -0.03 | -0.13 – 0.07 | 0.611 | -0.04 | -0.12 – 0.05 | 0.427 |
| **Random effects** |  |  |  |  |  |  |  |  |  |
| σ^2^ | 0.36 |  |  | 0.34 |  |  | 0.35 |  |  |
| τ_00_ _school_ | 0.01 |  |  | 0.01 |  |  | 0.00 |  |  |
| ICC | 0.02 |  |  | 0.02 |  |  | 0.01 |  |  |
| Marginal R^2^ / Conditional R^2^ | 0.000 / 0.021 |  |  | 0.059 / 0.075 |  |  | 0.062 / 0.070 |  |  |

**Note.** Bold values indicate statistical significance at p < 0.05.

**Supplementary Table 11.** Linear multilevel regression analyses for school belonging

|  | **Model 1** | | | **Model 2** | | | | **Model 3** | | | |
| --- | --- | --- | --- | --- | --- | --- | --- | --- | --- | --- | --- |
| *Predictors* | *β* | *CI* | *p* | | *β* | *CI* | *P* | | *β* | *CI* | *p* |
| (Intercept) | 3.40 | 3.21 – 3.59 | **<0.001** | | 3.26 | 3.01 – 3.51 | **<0.001** | | 3.26 | 3.01 – 3.51 | **<0.001** |
| Sex - female |  |  |  | | -0.01 | -0.10 – 0.09 | 0.891 | | -0.00 | -0.10 – 0.09 | 0.938 |
| Age |  |  |  | | 0.05 | 0.01 – 0.10 | **0.021** | | 0.05 | 0.01 – 0.10 | **0.017** |
| Educational track – havo |  |  |  | | 0.21 | 0.01 – 0.41 | **0.035** | | 0.09 | -0.17 – 0.36 | 0.489 |
| Educational track – vwo |  |  |  | | 0.29 | 0.10 – 0.48 | **0.003** | | 0.15 | -0.13 – 0.44 | 0.280 |
| Highest parental education - mid |  |  |  | | 0.15 | -0.05 – 0.35 | 0.150 | | 0.15 | -0.06 – 0.35 | 0.158 |
| Highest parental education - high |  |  |  | | 0.10 | -0.09 – 0.29 | 0.319 | | 0.09 | -0.11 – 0.28 | 0.383 |
| Migration background - yes |  |  |  | | -0.18 | -0.29 – -0.06 | **0.002** | | -0.18 | -0.29 – -0.06 | **0.002** |
| School socio-economic composition |  |  |  | |  |  |  | | -0.00 | -0.01 – 0.00 | 0.292 |
| School size - large |  |  |  | |  |  |  | | 0.18 | -0.11 – 0.47 | 0.230 |
| Smartphone ban type – full | 0.05 | -0.19 – 0.29 | 0.676 | | -0.00 | -0.21 – 0.20 | 0.991 | | -0.03 | -0.23 – 0.17 | 0.783 |
| **Random effects** |  |  |  | |  |  |  | |  |  |  |
| σ^2^ | 0.82 |  |  | | 0.82 |  |  | | 0.82 |  |  |
| τ_00_ _school_ | 0.06 |  |  | | 0.04 |  |  | | 0.03 |  |  |
| ICC | 0.07 |  |  | | 0.04 |  |  | | 0.04 |  |  |
| Marginal R^2^ / Conditional R^2^ | 0.001 / 0.071 |  |  | | 0.031 / 0.074 |  |  | | 0.039 / 0.077 |  |  |

**Note.** Bold values indicate statistical significance at p < 0.05.

**Supplementary Table 12.** Linear multilevel regression analyses for student-teacher connectedness

|  | **Model 1** | | | **Model 2** | | | | **Model 3** | | | |
| --- | --- | --- | --- | --- | --- | --- | --- | --- | --- | --- | --- |
| *Predictors* | *β* | *CI* | *p* | | *β* | *CI* | *P* | | *β* | *CI* | *p* |
| (Intercept) | 3.94 | 3.83 – 4.05 | **<0.001** | | 3.99 | 3.80 – 4.18 | **<0.001** | | 3.98 | 3.77 – 4.18 | **<0.001** |
| Sex - female |  |  |  | | -0.20 | -0.29 – -0.12 | **<0.001** | | -0.21 | -0.29 – -0.12 | **<0.001** |
| Age |  |  |  | | -0.03 | -0.06 – 0.01 | 0.103 | | -0.02 | -0.06 – 0.01 | 0.233 |
| Educational track – havo |  |  |  | | -0.03 | -0.17 – 0.10 | 0.623 | | 0.11 | -0.11 – 0.33 | 0.342 |
| Educational track – vwo |  |  |  | | -0.02 | -0.16 – 0.11 | 0.765 | | 0.13 | -0.11 – 0.37 | 0.289 |
| Highest parental education - mid |  |  |  | | 0.13 | -0.06 – 0.31 | 0.172 | | 0.14 | -0.05 – 0.32 | 0.146 |
| Highest parental education - high |  |  |  | | -0.02 | -0.19 – 0.15 | 0.811 | | -0.01 | -0.18 – 0.17 | 0.934 |
| Migration background - yes |  |  |  | | 0.09 | -0.01 – 0.19 | 0.080 | | 0.09 | -0.01 – 0.19 | 0.076 |
| School socio-economic composition |  |  |  | |  |  |  | | 0.00 | -0.00 – 0.00 | 0.555 |
| School size - large |  |  |  | |  |  |  | | -0.16 | -0.39 – 0.07 | 0.184 |
| Smartphone ban type – full | -0.17 | -0.31 – -0.03 | **0.016** | | -0.15 | -0.27 – -0.03 | **0.012** | | -0.14 | -0.29 – 0.01 | 0.068 |
| **Random effects** |  |  |  | |  |  |  | |  |  |  |
| σ^2^ | 0.67 |  |  | | 0.66 |  |  | | 0.66 |  |  |
| τ_00_ _school_ | 0.01 |  |  | | 0.01 |  |  | | 0.01 |  |  |
| ICC | 0.02 |  |  | | 0.01 |  |  | | 0.02 |  |  |
| Marginal R^2^ / Conditional R^2^ | 0.010 / 0.028 |  |  | | 0.033 / 0.041 |  |  | | 0.033 / 0.053 |  |  |

**Note.** Bold values indicate statistical significance at p < 0.05.

**Supplementary Table 13.** Linear multilevel regression analyses for classmate connectedness

|  | **Model 1** | | | **Model 2** | | | | **Model 3** | | | |
| --- | --- | --- | --- | --- | --- | --- | --- | --- | --- | --- | --- |
| *Predictors* | *β* | *CI* | *p* | | *β* | *CI* | *P* | | *β* | *CI* | *p* |
| (Intercept) | 3.49 | 3.30 – 3.67 | **<0.001** | | 3.45 | 3.20 – 3.70 | **<0.001** | | 3.45 | 3.20 – 3.70 | **<0.001** |
| Sex - female |  |  |  | | 0.03 | -0.07 – 0.13 | 0.572 | | 0.03 | -0.07 – 0.13 | 0.566 |
| Age |  |  |  | | 0.05 | 0.00 – 0.10 | **0.033** | | 0.05 | 0.00 – 0.10 | **0.031** |
| Educational track – havo |  |  |  | | 0.24 | 0.05 – 0.43 | **0.013** | | 0.20 | -0.07 – 0.47 | 0.153 |
| Educational track – vwo |  |  |  | | 0.39 | 0.20 – 0.58 | **<0.001** | | 0.34 | 0.05 – 0.63 | **0.023** |
| Highest parental education - mid |  |  |  | | 0.06 | -0.16 – 0.28 | 0.585 | | 0.06 | -0.16 – 0.28 | 0.591 |
| Highest parental education - high |  |  |  | | -0.09 | -0.29 – 0.12 | 0.407 | | -0.09 | -0.29 – 0.12 | 0.392 |
| Migration background - yes |  |  |  | | -0.24 | -0.36 – -0.12 | **<0.001** | | -0.24 | -0.36 – -0.12 | **<0.001** |
| School socio-economic composition |  |  |  | |  |  |  | | -0.00 | -0.00 – 0.00 | 0.859 |
| School size - large |  |  |  | |  |  |  | | 0.07 | -0.22 – 0.36 | 0.646 |
| Smartphone ban type – full | -0.01 | -0.25 – 0.22 | 0.921 | | -0.07 | -0.26 – 0.12 | 0.459 | | -0.08 | -0.27 – 0.12 | 0.426 |
| **Random effects** |  |  |  | |  |  |  | |  |  |  |
| σ^2^ | 0.94 |  |  | | 0.92 |  |  | | 0.92 |  |  |
| τ_00_ _school_ | 0.05 |  |  | | 0.03 |  |  | | 0.03 |  |  |
| ICC | 0.05 |  |  | | 0.03 |  |  | | 0.03 |  |  |
| Marginal R^2^ / Conditional R^2^ | 0.000 / 0.055 |  |  | | 0.037 / 0.065 |  |  | | 0.039 / 0.068 |  |  |

**Note.** Bold values indicate statistical significance at p < 0.05.

**Supplementary Table 14.** Linear multilevel regression analyses for bullying at school victimization

| *Predictors* | *β* | *CI* | *p* | *β* | *CI* | *P* | *β* | *CI* | *p* |
| --- | --- | --- | --- | --- | --- | --- | --- | --- | --- |
| (Intercept) | 1.20 | 1.12 – 1.29 | **<0.001** | 1.28 | 1.14 – 1.42 | **<0.001** | 1.30 | 1.15 – 1.45 | **<0.001** |
| Sex - female |  |  |  | -0.05 | -0.11 – 0.02 | 0.178 | -0.04 | -0.11 – 0.02 | 0.217 |
| Age |  |  |  | -0.01 | -0.04 – 0.01 | 0.363 | -0.01 | -0.04 – 0.01 | 0.334 |
| Educational track – havo |  |  |  | -0.10 | -0.20 – 0.01 | 0.064 | -0.04 | -0.20 – 0.13 | 0.669 |
| Educational track – vwo |  |  |  | -0.12 | -0.22 – -0.02 | **0.015** | -0.07 | -0.25 – 0.11 | 0.441 |
| Highest parental education - mid |  |  |  | -0.07 | -0.21 – 0.07 | 0.344 | -0.07 | -0.21 – 0.07 | 0.336 |
| Highest parental education - high |  |  |  | 0.03 | -0.10 – 0.16 | 0.639 | 0.02 | -0.12 – 0.15 | 0.785 |
| Migration background - yes |  |  |  | -0.05 | -0.12 – 0.03 | 0.238 | -0.04 | -0.12 – 0.03 | 0.273 |
| School socio-economic composition |  |  |  |  |  |  | -0.00 | -0.00 – 0.00 | 0.118 |
| School size - large |  |  |  |  |  |  | -0.07 | -0.24 – 0.09 | 0.392 |
| Smartphone ban type – full | 0.02 | -0.09 – 0.13 | 0.744 | 0.05 | -0.04 – 0.13 | 0.291 | 0.03 | -0.07 – 0.13 | 0.533 |
| **Random effects** |  |  |  |  |  |  |  |  |  |
| σ^2^ | 0.40 |  |  | 0.40 |  |  | 0.40 |  |  |
| τ_00_ _school_ | 0.01 |  |  | 0.00 |  |  | 0.00 |  |  |
| ICC | 0.02 |  |  | 0.00 |  |  | 0.01 |  |  |
| Marginal R^2^ / Conditional R^2^ | 0.000 / 0.019 |  |  | 0.013 / 0.018 |  |  | 0.021 / 0.032 |  |  |

**Note.** Bold values indicate statistical significance at p < 0.05.

**Supplementary Table 15.** Linear multilevel regression analyses for bullying at school perpetration

|  | **Model 1** | | | **Model 2** | | | | **Model 3** | | | |
| --- | --- | --- | --- | --- | --- | --- | --- | --- | --- | --- | --- |
| *Predictors* | *β* | *CI* | *p* | | *β* | *CI* | *P* | | *β* | *CI* | *p* |
| (Intercept) | 1.64 | 1.39 – 1.89 | **<0.001** | | 2.04 | 1.70 – 2.38 | **<0.001** | | 2.06 | 1.72 – 2.40 | **<0.001** |
| Sex - female |  |  |  | | -0.37 | -0.49 – -0.25 | **<0.001** | | -0.37 | -0.50 – -0.25 | **<0.001** |
| Age |  |  |  | | -0.03 | -0.09 – 0.03 | 0.354 | | -0.03 | -0.09 – 0.03 | 0.294 |
| Educational track – havo |  |  |  | | -0.05 | -0.31 – 0.21 | 0.711 | | 0.20 | -0.15 – 0.56 | 0.259 |
| Educational track – vwo |  |  |  | | -0.15 | -0.41 – 0.12 | 0.276 | | 0.14 | -0.23 – 0.51 | 0.461 |
| Highest parental education - mid |  |  |  | | -0.36 | -0.62 – -0.10 | **0.007** | | -0.36 | -0.62 – -0.09 | **0.008** |
| Highest parental education - high |  |  |  | | -0.25 | -0.49 – -0.00 | **0.048** | | -0.23 | -0.48 – 0.02 | 0.066 |
| Migration background - yes |  |  |  | | 0.16 | 0.02 – 0.31 | **0.028** | | 0.17 | 0.02 – 0.31 | **0.024** |
| School socio-economic composition |  |  |  | |  |  |  | | 0.00 | -0.00 – 0.01 | 0.418 |
| School size - large |  |  |  | |  |  |  | | -0.43 | -0.84 – -0.03 | **0.036** |
| Smartphone ban type – full | -0.07 | -0.39 – 0.25 | 0.661 | | -0.01 | -0.30 – 0.28 | 0.962 | | 0.05 | -0.25 – 0.34 | 0.750 |
| **Random effects** |  |  |  | |  |  |  | |  |  |  |
| σ^2^ | 1.39 |  |  | | 1.34 |  |  | | 1.34 |  |  |
| τ_00_ _school_ | 0.11 |  |  | | 0.08 |  |  | | 0.08 |  |  |
| ICC | 0.07 |  |  | | 0.06 |  |  | | 0.06 |  |  |
| Marginal R^2^ / Conditional R^2^ | 0.001 / 0.073 |  |  | | 0.035 / 0.092 |  |  | | 0.049 / 0.103 |  |  |

**Note.** Bold values indicate statistical significance at p < 0.05.

**Supplementary Table 16.** Linear multilevel regression analyses for cyberbullying victimization

|  | **Model 1** | | | **Model 2** | | | | **Model 3** | | | |
| --- | --- | --- | --- | --- | --- | --- | --- | --- | --- | --- | --- |
| *Predictors* | *β* | *CI* | *p* | | *β* | *CI* | *P* | | *β* | *CI* | *p* |
| (Intercept) | 1.50 | 1.21 – 1.80 | **<0.001** | | 1.86 | 1.52 – 2.19 | **<0.001** | | 1.91 | 1.57 – 2.25 | **<0.001** |
| Sex - female |  |  |  | | -0.20 | -0.29 – -0.10 | **<0.001** | | -0.20 | -0.29 – -0.10 | **<0.001** |
| Age |  |  |  | | -0.07 | -0.12 – -0.02 | **0.007** | | -0.07 | -0.12 – -0.02 | **0.005** |
| Educational track – havo |  |  |  | | -0.17 | -0.42 – 0.08 | 0.178 | | -0.00 | -0.31 – 0.30 | 0.982 |
| Educational track – vwo |  |  |  | | -0.20 | -0.45 – 0.05 | 0.115 | | -0.02 | -0.33 – 0.30 | 0.912 |
| Highest parental education - mid |  |  |  | | -0.31 | -0.51 – -0.10 | **0.004** | | -0.30 | -0.51 – -0.10 | **0.004** |
| Highest parental education - high |  |  |  | | -0.26 | -0.46 – -0.07 | **0.009** | | -0.25 | -0.45 – -0.06 | **0.012** |
| Migration background - yes |  |  |  | | 0.12 | 0.00 – 0.24 | **0.047** | | 0.12 | 0.00 – 0.24 | **0.043** |
| School socio-economic composition |  |  |  | |  |  |  | | 0.00 | -0.00 – 0.01 | 0.744 |
| School size - large |  |  |  | |  |  |  | | -0.40 | -0.80 – 0.01 | 0.057 |
| Smartphone ban type – full | -0.07 | -0.45 – 0.31 | 0.709 | | -0.02 | -0.35 – 0.31 | 0.896 | | 0.04 | -0.30 – 0.37 | 0.835 |
| **Random effects** |  |  |  | |  |  |  | |  |  |  |
| σ^2^ | 0.86 |  |  | | 0.85 |  |  | | 0.84 |  |  |
| τ_00_ _school_ | 0.18 |  |  | | 0.13 |  |  | | 0.13 |  |  |
| ICC | 0.17 |  |  | | 0.14 |  |  | | 0.13 |  |  |
| Marginal R^2^ / Conditional R^2^ | 0.001 / 0.175 |  |  | | 0.037 / 0.167 |  |  | | 0.066 / 0.189 |  |  |

**Note.** Bold values indicate statistical significance at p < 0.05.

**Supplementary Table 17.** Linear multilevel regression analyses for cyberbullying perpetration

|  | **Model 1** | | | **Model 2** | | | | **Model 3** | | | |
| --- | --- | --- | --- | --- | --- | --- | --- | --- | --- | --- | --- |
| *Predictors* | *β* | *CI* | *p* | | *β* | *CI* | *P* | | *β* | *CI* | *p* |
| (Intercept) | 1.51 | 1.28 – 1.74 | **<0.001** | | 1.98 | 1.68 – 2.28 | **<0.001** | | 1.98 | 1.68 – 2.29 | **<0.001** |
| Sex - female |  |  |  | | -0.31 | -0.41 – -0.20 | **<0.001** | | -0.31 | -0.41 – -0.20 | **<0.001** |
| Age |  |  |  | | -0.04 | -0.09 – 0.01 | 0.113 | | -0.04 | -0.09 – 0.01 | 0.104 |
| Educational track – havo |  |  |  | | -0.16 | -0.39 – 0.07 | 0.181 | | -0.06 | -0.37 – 0.26 | 0.727 |
| Educational track – vwo |  |  |  | | -0.23 | -0.46 – 0.00 | 0.054 | | -0.11 | -0.43 – 0.22 | 0.514 |
| Highest parental education - mid |  |  |  | | -0.44 | -0.67 – -0.21 | **<0.001** | | -0.44 | -0.66 – -0.21 | **<0.001** |
| Highest parental education - high |  |  |  | | -0.32 | -0.53 – -0.11 | **0.003** | | -0.31 | -0.52 – -0.10 | **0.005** |
| Migration background - yes |  |  |  | | 0.15 | 0.02 – 0.28 | **0.020** | | 0.15 | 0.02 – 0.28 | **0.019** |
| School socio-economic composition |  |  |  | |  |  |  | | 0.00 | -0.00 – 0.01 | 0.363 |
| School size - large |  |  |  | |  |  |  | | -0.17 | -0.53 – 0.19 | 0.362 |
| Smartphone ban type – full | -0.03 | -0.32 – 0.26 | 0.826 | | 0.04 | -0.22 – 0.30 | 0.766 | | 0.07 | -0.20 – 0.34 | 0.609 |
| **Random effects** |  |  |  | |  |  |  | |  |  |  |
| σ^2^ | 1.05 |  |  | | 1.01 |  |  | | 1.01 |  |  |
| τ_00_ _school_ | 0.09 |  |  | | 0.07 |  |  | | 0.07 |  |  |
| ICC | 0.08 |  |  | | 0.06 |  |  | | 0.07 |  |  |
| Marginal R^2^ / Conditional R^2^ | 0.000 / 0.083 |  |  | | 0.046 / 0.107 |  |  | | 0.053 / 0.115 |  |  |

**Note.** Bold values indicate statistical significance at p < 0.05.
